# Timed inhibition of CDC7 increases CRISPR-Cas9 mediated templated repair

**DOI:** 10.1038/s41467-020-15845-1

**Published:** 2020-04-30

**Authors:** Beeke Wienert, David N. Nguyen, Alexis Guenther, Sharon J. Feng, Melissa N. Locke, Stacia K. Wyman, Jiyung Shin, Katelynn R. Kazane, Georgia L. Gregory, Matthew A. M. Carter, Francis Wright, Bruce R. Conklin, Alex Marson, Chris D. Richardson, Jacob E. Corn

**Affiliations:** 1 Innovative Genomics Institute, University of California, Berkeley, CA 94703 USA; 2 Department of Molecular and Cell Biology, University of California, Berkeley, CA 94703 USA; 3 Gladstone Institutes, San Francisco, CA 94158 USA; 4 Department of Microbiology and Immunology, University of California, San Francisco, CA 94143 USA; 5 Diabetes Center, University of California, San Francisco, CA 94143 USA; 6 Department of Medicine, University of California, San Francisco, CA 94143 USA; 7 Department of Molecular, Cellular, and Developmental Biology, University of California, Santa Barbara, CA 93106 USA; 8 Department of Biology, Institute of Molecular Health Sciences, ETH Zürich, 8093 Zurich, Switzerland; 9 Departments of Medicine, Ophthalmology, and Pharmacology, University of California, San Francisco, CA 94143 USA; 10 Parker Institute for Cancer Immunotherapy, San Francisco, CA 94129 USA; 11 Chan Zuckerberg Biohub, San Francisco, CA 94158 USA

**Keywords:** Genetic engineering, CRISPR-Cas9 genome editing, Homologous recombination

## Abstract

Repair of double strand DNA breaks (DSBs) can result in gene disruption or gene modification via homology directed repair (HDR) from donor DNA. Altering cellular responses to DSBs may rebalance editing outcomes towards HDR and away from other repair outcomes. Here, we utilize a pooled CRISPR screen to define host cell involvement in HDR between a Cas9 DSB and a plasmid double stranded donor DNA (dsDonor). We find that the Fanconi Anemia (FA) pathway is required for dsDonor HDR and that other genes act to repress HDR. Small molecule inhibition of one of these repressors, CDC7, by XL413 and other inhibitors increases the efficiency of HDR by up to 3.5 fold in many contexts, including primary T cells. XL413 stimulates HDR during a reversible slowing of S-phase that is unexplored for Cas9-induced HDR. We anticipate that XL413 and other such rationally developed inhibitors will be useful tools for gene modification.

## Abstract (web summary)

Altering cellular responses to double-strand breaks in DNA could rebalance CRISPRediting outcomes. Here, the authors use a pooled CRISPR screen to identify inhibition of CDC7 as a strategy to improve HDR outcomes.

## Introduction

Genome editing with targeted nucleases, such as CRISPR-Cas9, is a powerful tool for research and a promising approach for therapeutic treatment of human disease. One strategy for efficient genome editing in eukaryotic cells introduces a ribonucleotide protein (RNP) complex comprised of the type II endonuclease Cas9 and a guide RNA (gRNA), which create a double strand DNA break (DSB) at a targeted location in the genome^1,2^. This DSB is repaired by cellular DNA repair pathways to produce two outcomes: error-prone sequence disruption by insertion or deletion (indels) at the DSB, or precise sequence modification via homology directed repair (HDR) that copies donor DNA sequences into the DSB. The targeted incorporation of exogenous DNA sequences enables multiple new research avenues in metazoan cells, including endogenous epitope tagging and the insertion of SNPs to test disease causation, and using these techniques in human tissues could enable therapeutics to correct genetic lesions that drive human disease^3,4^. Strategies to favor precise HDR outcomes over deleterious error-prone repair in human cells are therefore of intense interest both to improve understanding of biological pathways and enable new therapeutic options.

Human cells have multiple overlapping DSB repair pathways that have been implicated in Cas9-mediated gene modification^5–7^ such as alternative-end joining, synthesis-dependent strand annealing, and HDR, which encompasses multiple different mechanisms of repair. To investigate the various HDR mechanisms in greater detail, we previously developed a reporter^8^ that allowed us to interrogate the genetic requirements of Cas9-mediated HDR using single-stranded donor DNA (ssDonor) and discovered that single strand template repair (SSTR) requires the Fanconi Anemia (FA) DNA repair pathway^9^. We furthermore found that SSTR does not depend on the classic RAD51 pathway, unlike homologous recombination (HR) repair from a double stranded DNA donor (dsDonor). These distinct requirements for HDR from ssDonor and dsDonor implied that different donors produce molecularly identical gene modifications via different mechanistic routes. To more completely map how different types of donors mediate Cas9-induced HDR, we used genetic screening to reveal the DNA repair factors that are involved in HR using a dsDonor. Here, we describe genes that upregulate or downregulate HR from dsDonor templates, finding pathways that are both shared with and distinct from SSTR. We furthermore investigate factors whose knockdown increases Cas9-induced HDR, discovering that timed administration of a small molecule inhibitor of one of these factors, cycle 7-related protein kinase (CDC7), increases HR and SSTR by up to 3.5-fold in multiple contexts including primary human T cells.

## Results

### Cas9-induced SSTR and HR overlap, with key distinctions

**Fig. 1:**
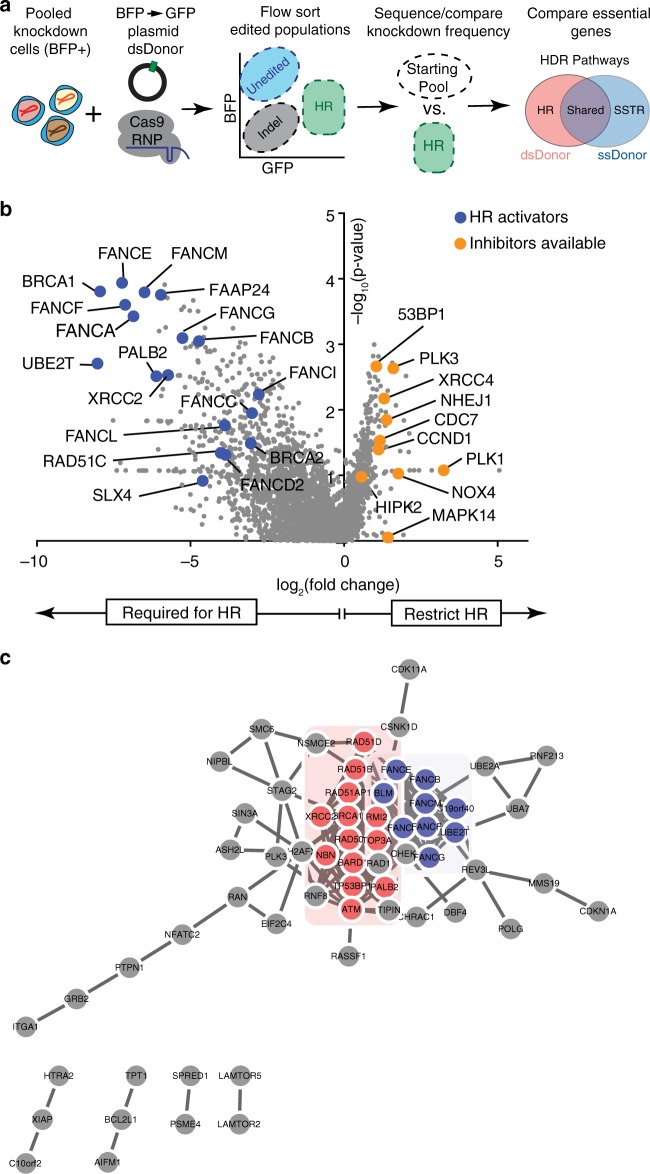
A pooled CRISPR screen reveals pathways that regulate templated repair using Cas9-RNP and a plasmid dsDonor. **a** Schematic showing BFP → GFP CRISPRi screening strategy. Pooled K562-CRISPRi cells that stably express BFP and a library of gRNAs targeting DNA metabolism genes are further edited with Cas9-RNP that cuts within *BFP* and a plasmid dsDonor template that contains a promoterless copy of *GFP*. Cas9 gene editing results in three populations: Unedited (BFP^+^GFP−), Indel (BFP−GFP−), and HR (BFP−GFP^+^). Guide RNA frequencies in the HR population were quantified and compared to the unsorted population to identify genes that promote/restrict HR. These genes were compared to data from a prior screen using ssDNA donor^8^. **b** Genes regulating dsDonor HR. A volcano plot of pooled screen hits showing the Fanconi Anemia pathway (blue) and HR repressors with available small molecule inhibitors (orange). Data presented from *n* = 2 screen replicates. **c** Genetic regulators of HR participate in physical interactions with one another. Significant (*p* < 0.005, Mann–Whitney test) hits that impacted HR were cross-referenced with the STRING protein interaction database^12^. Factors involved in homologous recombination (HR, red) or Fanconi Anemia (FA, blue) pathways were identified by GO term. Only regulators appearing in the screen and having at least one interaction with another screening hit are shown.

We adapted a previously described pooled screening platform^9^ to define the individual contribution made by each of thousands of genes to Cas9-mediated gene replacement using a dsDonor plasmid template. The basis of this platform is the stable expression of three components in each cell: (1) a dCas9-KRAB CRISPRi construct^10^, (2) a *BFP* reporter gene^8^, and (3) a gRNA targeting the transcription start site (TSS) of a single gene. We constructed a gRNA library to target genes with Gene Ontology (GO) terms related to *DNA*, comprising a library of approximately 2000 genes at a density of five guides per TSS^11^. Pooled K562 erythroleukemia cell populations stably expressing BFP and individually downregulating a specific gene were transiently nucleofected with a Cas9 RNP targeted to introduce a DSB in the *BFP* reporter, together with a dsDonor plasmid with a *GFP* sequence template that converts BFP to GFP upon successful HR^8^ (Fig. 1a). Edited cell populations were separated by fluorescence-activated cell sorting (FACS) (HR: BFP^−^GFP^+^; gene disruption: BFP^−^GFP^−^) (Supplementary Fig. 1a), and gRNA frequency in each population was determined by sequencing the stably integrated gRNA cassette. Genes whose upregulation and downregulation altered each repair outcome were determined by comparing the sorted populations to the edited but unsorted cell population. Similarities between the reagents and techniques used in this screening approach permitted direct comparison with our earlier screen editing the same locus but utilizing a ssDonor^9^ (Fig. 1a).

Guide RNAs targeting genes that restrict HR were enriched in the BFP^−^ GFP^+^ population (because their knockdown favors HR), while gRNAs targeting genes that are required for HR were depleted in the BFP^−^ GFP^+^ population (Fig. 1b). As expected, classic HR factors such as BRCA1, BARD1, RAD50, and NBN were genetically required for Cas9-mediated repair from a plasmid dsDonor, and these often were found in the same physical complexes with one another in the STRING protein database^12^ (Fig. 1c). Our prior work defined the Fanconi Anemia (FA) pathway as necessary for Cas9-mediated SSTR^9^, and we now report that almost the entire FA repair pathway is also required for Cas9-induced HR. 31 of 40 FA and FA-related genes were required for HR, suggesting that this is an activity of the overall FA pathway (Supplementary Fig. 1b) and indicating its importance for all forms of Cas9-mediated HDR^13^.

The shared reliance of Cas9-induced SSTR and HR on the FA pathway motivated us to systematically explore the overlapping genetic dependencies of HDR from single and double stranded templates. We performed GO term analysis^14^ on statistically significant (*p* < 0.05) genes involved in SSTR and HR to define pathways involved in each process (Supplementary Fig. 1c and Supplementary Data 1 GO Terms). There was substantial overlap between SSTR and HR: both pathways require Fanconi Anemia Repair, nucleotide excision repair (NER), and strand displacement activities, which is driven by mutual reliance on FA proteins, members of the TFIIH complex, and the BLM helicase. Both SSTR and HR may therefore challenge cells to balance NER-like single strand editing activities with templated repair events, as has been suggested for repair of interstrand crosslinks^15^. Despite these similarities, SSTR and HR are distinct in notable ways. SSTR but not HR is associated with Negative regulation of transposition, a GO term including our SSTR hits APOBEC3C, D, F, and G. Originally reported as RNA editing enzymes, these enzymes are known to modify ssDNA during gene editing reactions^16^, and similar proteins have been repurposed as targeted DNA base-editing reagents^17^. HR on the other hand depends on genes involved in Double Strand Break Repair via Homologous Recombination, which includes factors that potentiate meiotic homologous recombination (Supplementary Fig. 1c). These observations suggest that all HDR requires the FA pathway, but the HR and SSTR mechanisms each require specialized activities to respond to donor topologies or intermediate structures specific to each repair process.

We identified several genes that normally repress HR, such that their knockdown enhances HR efficiency (Fig. 1b). Some of these genes are consistent with a model in which NHEJ and HR compete to repair DSBs, so inhibition of one pathway favors the other^18–20^. Examples of these repressor genes include TP53-binding protein 1 (53BP1), X-ray repair cross-complementing protein 4 (XRCC4) and non-homologous end joining factor 1 (NHEJ1), which interact at DSBs to promote DNA ligase 4 (LIG4) association during non-homologous end-joining (NHEJ)^21^. Other repressors identified here have not previously been reported to increase HR efficiency, but have roles in processes that have been linked to DNA repair outcomes such as cell cycle progression. These proteins include CDC7, polo-like kinase 1 (PLK1), E2F transcription factor 1 (E2F1), and polo-like kinase 3 (PLK3).

### Inhibiting HR repressors increases both HR and SSTR

HR is quite inefficient in human cells yet desirable for its ability to precisely engineer genomic sequences and even insert long (>500 bp) sequences such as epitope tags or ORF replacements^22^. Our exploration of genes that regulate HR presented us with a number of candidate repressors whose knockdown would increase HR efficiency. However, gene editing is frequently performed in primary cell types or other experimental contexts where transcriptional or genetic repression is unsuitable. Thus, small molecule treatments that increase HR would be more desirable because they can be administered in a variety of settings and easily withdrawn. We selected genes that repress SSTR, HR, or both and performed an extensive literature search to find small molecule inhibitors of these HDR repressors (Fig. 1b). We focused on eight commercially available small molecules that are reported to inhibit CCND1, CDC7, HIPK2, MAPK14, NOX4, PLK3, PLK1, and 53BP1 (Supplementary Fig. 1d).

**Fig. 2:**
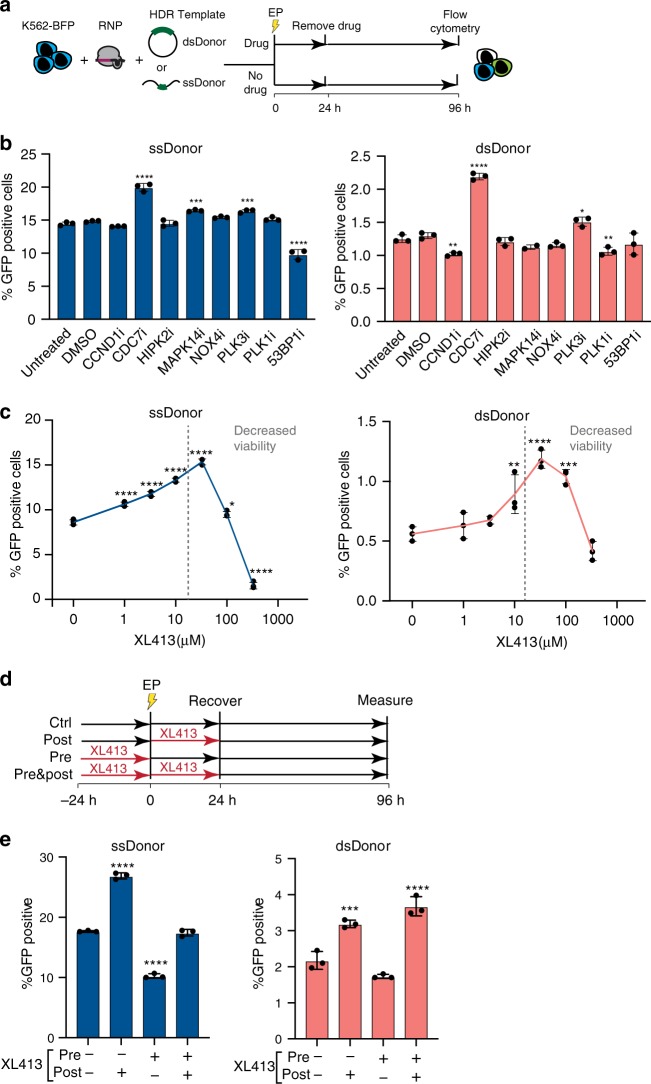
Enhancing HDR by small molecule inhibition of factors discovered in genetic screening. **a** Schematic of small molecule evaluation. K562-BFP cells were nucleofected with Cas9-RNPs targeting the *BFP* transgene and either plasmid dsDonor or oligonucleotide ssDonor templates. After electroporation (EP), cells were added to media with or without compound. Cell populations were recovered into fresh media after 24 h and analyzed by flow cytometry after 96 h. **b** CDC7 inhibition with XL413 significantly increases SSTR and HR. Shown is the percentage of GFP-positive cells by flow cytometric analysis of K562-BFP cell populations 4 days post nucleofection with ssDonor (left) or dsDonor (right) comparing different chemical compound treatments. *X*-axis indicates the intended molecular target of the small molecule inhibitors. **c** XL413 increases HDR in a concentration-dependent manner for both SSTR and HR pathways. Shown is the percentage of GFP positive cells 4 days post nucleofection for editing with ssDonor (left) and plasmid dsDonor (right). Dashed line indicates point where XL413 toxicity becomes significant. **d** Schematic showing strategies for pre-treatment and post-treatment with XL413. **e** Editing outcomes in K562-BFP depend on timing of XL413 administration. Cells were untreated, treated for 24 h with XL413 before nucleofection (pre), treated for 24 h with XL413 after nucleofection (post) or both (pre- and post). Percentage of GFP positive cells was determined 4 days post nucleofection using flow cytometry. All values are shown as mean ± SD (*n* = 3 biological replicates). Statistical significances were calculated by ordinary one-way ANOVA and Dunnet’s multiple comparison test comparing different treatments to the control (adjusted *p*-values are reported as *p < 0.05, **p < 0.01, ***p < 0.001, ****p < 0.0001). Source data are available in the Source Data file.

We first asked if chemical inhibition of these HR repressors affects HR or SSTR editing outcomes using our K562 cell line stably expressing the BFP-to-GFP HDR reporter system^8^. Reporter cells were nucleofected with a Cas9 RNP targeting the *BFP* reporter gene and either a ssDonor or plasmid dsDonor. We reasoned that small molecule inhibition of HR repressors would be most effective during gene editing (e.g., post-treatment), so we treated cells with different inhibitors for 24 h and then recovered in inhibitor-free media (Fig. 2a). BFP-to-GFP HDR outcomes were monitored by flow cytometry after four days (Supplementary Fig. 2a). Many compounds resulted in no change or even a reduction of HR, which could be caused by impaired cell fitness. Inhibition of mitogen-activated protein kinase 14 (MAPK14) with SB220025 slightly enhanced SSTR (1.1-fold), and inhibition of PLK3 with GW843682X slightly increased both SSTR and HR from the plasmid dsDonor (1.1-fold and 1.2-fold).

By contrast, treatment with the CDC7 inhibitor XL413^23^ resulted in a significant increase in both SSTR and HR (1.4-fold and 1.8-fold, *p* < 0.0001) (Fig. 2b). siRNA inhibition of CDC7 was less effective at promoting HR (1.4-fold) than small molecule inhibition (Supplementary Fig. 2b, c), which suggests that inactivating CDC7 kinase activity may be more effective than reducing its overall levels. The effect of XL413 is concentration dependent, as both SSTR and HR increase in a dose-dependent manner, with 33 μM XL413 increasing HDR 1.8-fold to 2.1-fold (Fig. 2c). XL413 concentrations up to 10 μM and exposure for up to 24 h did not result in a notable decrease in viability in K562 cells (Supplementary Fig. 2d, e), and we therefore used 10 μM XL413 in subsequent experiments unless noted otherwise. To optimize CDC7 inhibition, we explored if different timing of exposure to XL413 (e.g., pre-treatment) altered HDR rates. However, pre-exposure to XL413 and then release during editing resulted in reduced levels of HDR, while pre-exposure and post-exposure did not significantly increase HDR over post-treatment alone (Fig. 2d, e). We therefore conclude that the optimal timing for HDR is to incubate edited cells immediately post-treatment in media containing CDC7 inhibitors.

### CDC7 inhibition increases HDR at multiple endogenous loci

**Fig. 3:**
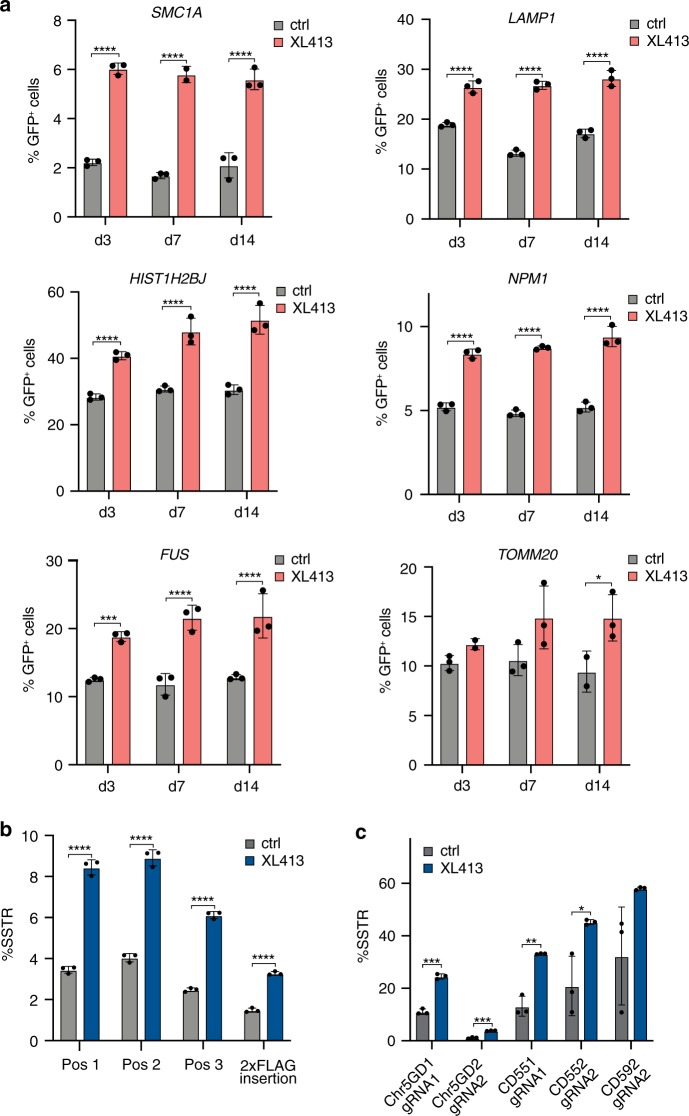
CDC7 inhibition increases HR and SSTR of diverse genetic cargoes in cell lines. **a** XL413 increases HR at endogenous loci with large HR donors. K562 cells were nucleofected with Cas9-RNPs targeting six different genomic loci (*SMC1A, LAMP1, NPM1, HIST2H2BJ, FUS*, and *TOMM20*) with plasmid dsDonor to knock-in a *GFP* sequence to the C-terminal end of the gene. Half of the pool of nucleofected cells was treated with 10 μM XL413 for 24 h while the other half remained untreated. Flow cytometric analysis determined the percentage of GFP positive cells 3, 7, and 14 days after nucleofection. Gating strategy depicted in Supplementary Fig. 3a. **b** XL413 increases SSTR at endogenous loci. K562 cells were nucleofected with RNP targeting *TOMM20* and an ssDonor encoding 2xFLAG (Supplementary Fig. 3b) in the presence or absence of 10 μM XL413 for 24 h, gDNA was extracted after 4 days, and SSTR frequencies were determined by amplicon sequencing. **c** XL413 increases the frequency of SNP conversion. RNPs targeting five loci and ssDonors encoding SNPs were introduced into cells and editing outcomes quantified as described in **b**. All values are shown as mean ± SD (*n* = 3 biological replicates). Statistical significances were calculated by unpaired two-tailed *t*-test using the Holm-Sidak method (p-values are reported as *p < 0.05, **p < 0.01, ***p < 0.001, ****p < 0.0001) Source data are available in the Source Data file.

We next asked if XL413’s ability to stimulate HDR is generally applicable to multiple genomic loci and HDR cargo sizes. We used Cas9-induced HR from a plasmid dsDonor template to knock-in a *GFP* coding sequence at the C-terminus of various genes in K562 cells using editing reagents previously developed as part of a comprehensive cell-tagging effort^24^: *Lysosomal-associated membrane protein 1 (LAMP1), fibrillarin (FBL), histone H2BJ (HIST1H2BJ), nucleophosmin (NPM1), Structural maintenance of chromosomes protein 1* *A (SMC1A), RNA binding protein FUS (FUS), and translocase of outer mitochondrial membrane 20 (TOMM20)*. We found that treatment with XL413 for 24 h immediately after nucleofection increased the HR efficiency at all loci, ranging from 1.6-fold to 3.5-fold irrespective of the original frequency of HR (Fig. 3a and Supplementary Fig. 3a). Importantly, the frequency of HR remained unchanged over two weeks of cell culture, indicating that edits produced during XL413 treatment are stable over long time periods.

We then investigated if SSTR is similarly increased by CDC7 inhibition at endogenous loci. We designed two ssDonor editing strategies: insertions of a 2 × FLAG-tag at the C-terminus of *TOMM20* (Supplementary Fig. 3b), and SNP modifications at five different genomic loci. Using amplicon PCR and next-generation sequencing, we found that XL413-treated K562 cells had up to a 2.5-fold increase in SSTR-based FLAG tagging and introduction of SNPs relative to untreated cells (Fig. 3b, c and Supplementary Data 2). Genomic increases in tagging corresponded with increased ability to detect FLAG-tagged TOMM20 by Western blotting (Supplementary Fig. 3c, d). These findings suggest that CDC7 inhibition robustly increases SSTR and HR, and can be used to increase the frequency of both single nucleotide substitutions and larger endogenous gene tagging.

### CDC7 inhibition enhances HDR in primary cells

**Fig. 4:**
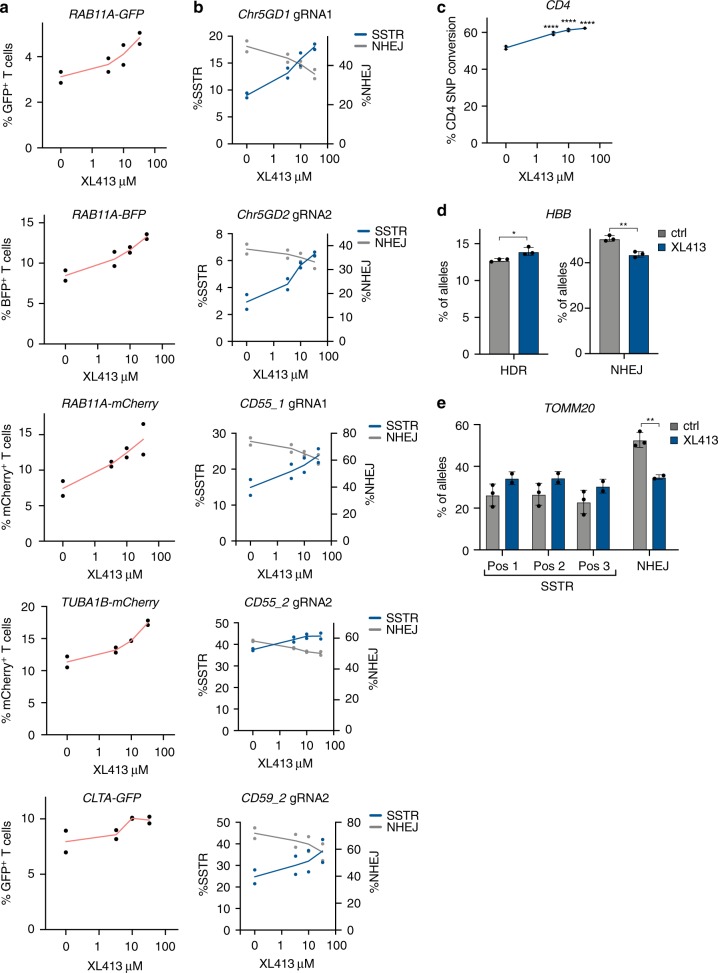
CDC7 inhibition increases HR and SSTR in primary human cells. **a** XL413 increases HR at endogenous loci in T cells. Primary CD3^+^ T cells from two healthy blood donors were nucleofected with RNPs targeting three different genomic loci (*RAB11A, TUBA1B*, and *CLTA*) with a linear dsDonor template to knock-in a fluorescent reporter protein fused to the N-terminal end of the target protein. Nucleofected cells were treated with the indicated doses of XL413 for 24 h, then the percentage of reporter-positive T cells was quantified by flow cytometry 4 days after nucleofection. Gating strategy is depicted in (Supplementary Fig. 4a). **b** XL413 increases SSTR at endogenous loci. Primary CD3^+^ T cells were nucleofected with RNPs targeting the indicated loci with ssDonors to insert point mutations, treated with the indicated concentration of XL413 for 24 h. After 4 days, gDNA was extracted and SSTR and NHEJ frequencies determined by amplicon sequencing. **c** XL413 increases the frequency of SNP conversion. RNPs targeting the CD4 locus and an ssDonor encoding a naturally occurring SNP (Supplementary Fig. 4b–d) were introduced into T cells from three donors, and editing outcomes quantified by flow cytometry after 4 days. Statistical significances were calculated by ordinary one-way ANOVA and Dunnet’s multiple comparison test (adjusted *p*-values are reported as *p < 0.05, **p < 0.01, ***p < 0.001, ****p < 0.0001). **d** XL413 increases the frequency of SNP conversion in the *HBB* gene in primary human CD34^+^ HSPCs. RNPs targeting the *HBB* locus and an ssDonor encoding the E6V mutation^25^ were nucleofected into HSPCs in the presence or absence of 30 μM XL413, gDNA was extracted after 4 days, and mutation frequencies determined by amplicon sequencing. Statistical significances were calculated by unpaired two-tailed *t*-test using the Holm-Sidak method (*p*-values are reported as *p < 0.05, **p < 0.01, ***p < 0.001, ****p < 0.0001). **e** XL413 increases the frequency of SSTR in primary human CD34^+^ HSPCs. Cells were nucleofected with RNPs targeting *TOMM20* with a ssDonor that contains three synonymous SNPs in the presence or absence of 30 μM XL413 for 24 h, gDNA was extracted after 4 days, and SSTR and NHEJ frequencies were determined by amplicon sequencing. Values are shown as mean ± SD of the indicated number of samples. Source data are available in the Source Data file.

HDR in primary cells is a long-standing goal of gene editing, both for its ability to correct disease-causing SNPs and to deliver large payloads such as chimeric antigen receptors^22,25^. We therefore investigated the ability of XL413 to increase HR in human T cells derived from peripheral blood mononuclear cells (PBMCs) of healthy donors. We performed gene editing using RNPs targeting the *RAB11A*, *TUBA1B*, and *CLTA* loci, together with linear dsDonor templates for each locus to generate fluorescently-tagged protein fusions (GFP, mCherry, or BFP)^22^. XL413 treatment after editing produced a dose-dependent increase in HR efficiency at each locus in T cells (Fig. 4a), without evidence of decreased viability (Supplementary Fig. 4a). Using a ssDonor template and targeting five additional genomic loci, we found that XL413 also boosts SSTR in human T cells while NHEJ frequencies decreased (Fig. 4b and Supplementary Data 2). This shows that CDC7 inhibition does not affect the total amount of editing but rather shifts the ratio of edited alleles from NHEJ to HDR. Furthermore, treatment with XL413 increased SSTR beyond 60% efficiency when introducing a naturally occurring SNP that alters recognition of CD4 by the OKT4 antibody without affecting recognition by the SK3 antibody^26^ (Fig. 4c and Supplementary Fig. 4b–d).

Reversal of pathogenic mutations in patient-derived stem cells is a promising therapeutic application of HDR^27,28^. We therefore tested XL413 in primary human hematopoietic stem and progenitor cells (HSPCs). We found that XL413 addition during SSTR editing in HSPCs slightly elevated SNP conversion at two different loci, including the causative allele of sickle cell disease (Fig. 4d, e and Supplementary Data 2). Short-term HSPC viability was not grossly decreased by treatment with XL413 (Supplementary Fig. 4e). Taken together, we find that XL413 can increase multiple forms of HDR in two primary cell types. Additional work will be needed to determine the long-term effects of XL413 treatment to increase the edited fraction of cells if deployed in a pre-clinical context.

### CDC7 inhibition promotes homozygous HDR

**Fig. 5:**
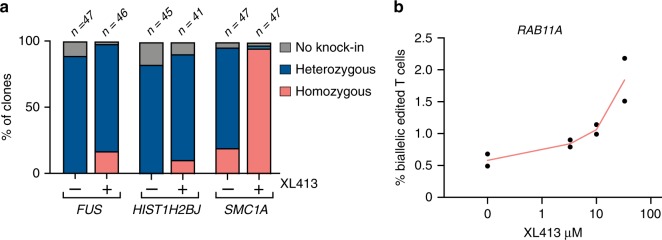
XL413 increases biallelic editing at endogenous loci. **a** XL413 increases homozygous editing in K562 cells. Cells were nucleofected with RNPs targeting three different genomic loci (*FUS, HIST1H2BJ*, and *SMC1A*) and plasmid dsDonor to knock a GFP reporter protein into the C-terminal end of the gene. Nucleofected cells were untreated or treated with 10 μM XL413 for 24 h. GFP^+^ cells were single-cell sorted 4 days post nucleofection, clonally expanded, and allelic editing frequencies quantified by junction PCR (Supplementary Fig. 5a, b). The total number of clones analyzed is noted above each column with the proportion of heterozygous, homozygous, or no knock-in (GFP^+^ but no detection of GFP sequence at on-target site) genotype shown per condition. **b** XL413 increases biallelic editing in T cells. CD3^+^ T cells were nucleofected with RNPs targeting the *RAB11A* locus and two linear dsDonors encoding N-terminal fusions of either mCherry or BFP and treated with the indicated concentrations of XL413 for 24 h. Single-fluorescent or double-fluorescent reporter populations were quantified by flow cytometry (Supplementary Fig. 5c, d). Shown is the frequency of biallelic dual-positive integration (BFP^+^/mCherry^+^) events displayed as mean ± SD (*n* = 2 biological replicates). Source data are available in the Source Data file.

Having tested the ability of XL413 to increase the fraction of edited alleles in a bulk population, we next evaluated whether XL413 increases the frequency of multi-allelic editing on a per-cell basis. We performed GFP-tagging in K562 cells at multiple loci as previously described with or without XL413, fluorescently sorted single GFP-expressing clones, and quantified knock-in alleles in each clone by junction PCR (Supplementary Fig. 5a, b). We found that XL413 dramatically increased the frequency of homozygous HR even in triploid K562 cells from undetectable to approximately 17% of cells edited at the *FUS* and *HIST1H2BJ* loci. HDR at the *SMC1A* locus was increased 4.9-fold such that almost all clones tested were homozygous (Fig. 5a). Homozygous editing was also increased up to 3.2-fold in T cells from healthy donor PBMCs, as measured by a fluorescent reporter assay that mixes two linear dsDonor templates with different fluorophores (BFP or mCherry) both targeted to the *RAB11A* locus^22^ (Fig. 5b and Supplementary Fig. 5c, d). Our T-cell homozygous HDR assay only measures integration of the two different fluorophores at a given locus (e.g., BFP^+^/mCherry^+^), and so these values likely undercount the true frequency of biallelic editing by missing dual integration of the same fluorophore (e.g., BFP^+^/BFP^+^). We therefore conclude that CDC7 inhibition increases the rate of homozygous HDR, which could be very useful for both research and therapeutic purposes.

### CDC7 inhibition boosts HDR in multiple cell types

We profiled XL413 in additional cell types at multiple loci, and found that cells of different origins vary in the ability of XL413 to increase HDR (Supplementary Fig. 6a). K562 cells, T cells, and HSPCs are responsive to XL413, boosting all tested forms of HDR up to 3.5-fold. Using XL413 in HEK293T, U-251, HeLa and induced pluripotent stem cells (iPSCs) increased HDR at some loci and with some donors, but not with others (Supplementary Fig. 6a, b and Supplementary Data 2). XL413 did not increase HDR in primary mouse glial cultures, primary human dermal lymph endothelial cells (HDLECs) and primary human dermal fibroblasts (HDFs). However, these cell types proliferate particularly slowly and CDC7 inhibition may not work in this context (Supplementary Fig. 6a, c). Because XL413 is known to be non-functional in a cell-type specific manner^29^, we tested other CDC7 inhibitors for their ability to increase HDR in contexts where XL413 was inactive. The dual CDC7/CDK9 inhibitor PHA-767491^30^ increased HR in K562 cells 2.3-fold and at two additional loci (1.5-fold and 1.7-fold) in HCT116 cells, which do not respond consistently to XL413 (Supplementary Fig. 7a, b). However, we found that treatment with PHA-767491 is more toxic to cells than XL413 and that it results in a distinctly different cell cycle profile than XL413 (Supplementary Fig. 7c–e). Overall, we find that transcriptional inhibition of CDC7 (Supplementary Fig. 2b, c) and two small molecule inhibitors of CDC7 can all increase levels of HDR during Cas9 genome editing.

### XL413 is more effective than other chemical HDR enhancers

**Fig. 6:**
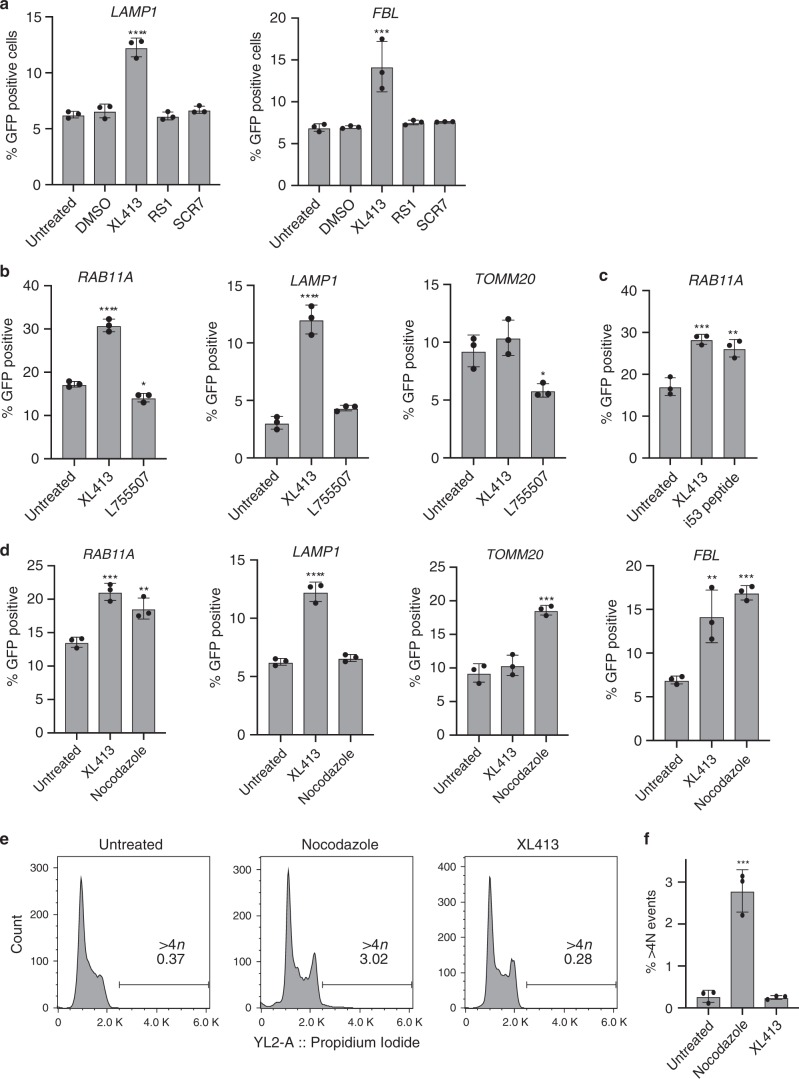
Comparisons between XL413 and other small molecule HDR-boosting strategies in K562 cells. **a** Direct comparison between XL413 and RS1 or SCR7. Cells were nucleofected with RNP targeting the *LAMP1* or *FBL* loci with dsDonors encoding C-terminal fusions to GFP, cultured in XL413 (33 μM), SCR7 (1 μM) or RS-1 (10 μM) for 24 h, and editing rates were measured 4 days post nucleofection by flow cytometry. **b** Comparison between XL413 and L755507. Cells were nucleofected with RNPs targeting the *RAB11A*, *LAMP1*, or *TOMM20* loci with dsDonors encoding N-terminal (*RAB11A*) or C-terminal (*LAMP1*, *TOMM20*) fusions to GFP, cultured in XL413 (10 μM) or L755507 (5 μM) for 24 h, and editing rates were measured 4 days post nucleofection by flow cytometry. **c** Comparison between XL413 and i53. Cells were nucleofected with RNP targeting the *RAB11A* locus with dsDonor encoding an N-terminal fusion to *GFP*. i53-treated samples were lipofected with (3 μg) plasmid encoding i53 and BFP 24 h prior to nucleofection with editing reagents. XL413-treated samples were cultured in 10 μM XL413 for 24 h after nucleofection, then editing outcomes were measured 4 days post-nucleofection by flow cytometry. i53-transfected cells were gated using a BFP fluorescent marker expressed from the i53 expression plasmid. **d** Direct comparison between XL413 and nocodazole. Cells were nucleofected with RNP targeting the *RAB11A, LAMP1, TOMM20*, or *FBL* loci with dsDonors encoding N-terminal (*RAB11A*) or C-terminal (*LAMP1, TOMM20*, and *FBL*) fusions to GFP. Samples were treated with nocodazole (33 ng mL^−1^) for 24 h before nucleofection. XL413-treated samples were cultured in 10 μM XL413 for 24 h after nucleofection. Editing rates were measured 4 days post-nucleofection by flow cytometry. **e** 1Cells were treated with nocodazole or XL413 for 24 h and then released into normal media for four days prior to propidium iodide staining and flow cytometry. Example plots are shown for untreated, nocodazole, and XL413 treated samples. **f** Quantification of frequency of cells with *a* > 4 N DNA content (indicative of failed mitoses) from **e**. All values are shown as mean ± SD (*n* = 3 biological replicates). Statistical significances were calculated by ordinary one-way ANOVA and Dunnet’s multiple comparison test (adjusted p-values are reported as *p < 0.05, **p < 0.01, ***p < 0.001, ****p < 0.0001). Source data are available in the Source Data file.

To contextualize the effectiveness of CDC7 inhibition, we compared XL413 to other approaches previously reported to boost HR. SCR7 inhibits NHEJ to rebalance DNA repair outcomes^20,31^, RS-1 is a RAD51 agonist that activates recombination^32,33^, L755507 is a β-3 adrenergic receptor agonist that works through an unknown mechanism^34^, and i53 inhibits 53BP1 to reduce NHEJ and favor recombination^35^. In our editing workflow, SCR7, RS-1, and L755507 had little effect on HR (Fig. 6a, b). Combining SCR7 or RS-1 with XL413 for 24 h also did not increase HR beyond XL413 alone (Supplementary Fig. 8a) i53 peptide treatment increased HR 1.5-fold when considering only the cells expressing the plasmid encoding for i53 (Fig. 6c), which is comparable to the 1.7-fold seen with XL413 in this experiment. However, when determining the efficiency of i53 to increase HR in the whole population of cells (Supplementary Fig. 8b) it is less effective than XL413, and does not further increase editing in combination with XL413 (Supplementary Fig. 8c).

### XL413 treatment produces a transient cell cycle arrest

Exposure of cells to CDC7 inhibitors, like XL413, has additional effects on cells beyond increasing HDR. CDC7 is the catalytically active subunit of DBF4-dependent kinase (DDK), which phosphorylates and activates the MCM helicase to initiate the G1/S transition^36,37^. XL413 has been reported to rapidly inhibit phosphorylation of MCM2 and arrest cells in early S-phase^23,38^. As timing of XL413 administration is important for its activity (Fig. 2d, e), we investigated the overlap between XL413 stimulation of HDR increase and cell cycle arrest. 24 h of XL413 treatment causes inhibition of MCM2 phosphorylation as measured by Western blot (Supplementary Fig. 9a) and cell cycle arrest phenotypes as measured using a FUCCI live cell reporter system^39^ and Hoechst33342 staining (Supplementary Fig. 9b–g). Importantly, these effects are reversible and return to baseline 24 h after XL413 is removed. Post-editing treatment with XL413 for 24 h therefore synchronizes cells in S phase, and we speculate that this extends the time cells spend in the HDR-permissive S/G2/M phases of the cell cycle. Such a model would also explain why dosing with XL413 before editing does not increase HDR, as the cell cycle delay occurs before editing reagents are introduced. Interestingly, treatment with other S-phase inhibitors such as Aphidicolin or Hydroxyurea did not increase HR consistently (Supplementary Fig. 9h) suggesting that cell cycle arrest with XL413 is distinct from arrest with other S phase inhibitors.

The microtubule poison nocodazole has been previously described to boost HDR by placing cells at a permissive point in the cell cycle^7^, and we next compared nocodazole treatment to XL413. In our hands, nocodazole increased HDR to a comparable level as XL413 when cells were arrested with nocodazole prior to editing (Fig. 6d). However, nocodazole has also been reported to increase the frequency of aneuploidy and failed mitoses^40–42^. We measured the frequency of cells with >4 N DNA content produced by failed mitoses in compound-treated and untreated cell cultures. We observed no significant increase in >4 N DNA staining in untreated or XL413 treated cells after four days, but we found a significant increase in these events with nocodazole treated samples (*p* < 0.001) (Fig. 6e, f).

In summary, we mined our HR and SSTR genetic dependency datasets to identify CDC7 inhibition as a potent strategy to boost HDR in human cell lines. XL413, a CDC7 inhibitor, improves the efficiency of gene editing workflows for basic research or therapeutic applications, increases gene replacement in multiple cell types, and has utility in challenging contexts with therapeutic potential such as primary T cells and HSPCs. CDC7 inhibition is most effective when administered while editing is underway and is thus maximally compatible with RNP-mediated gene editing workflows.

## Discussion

Our focused CRISPRi screen defined a network of genes that contribute to Cas9-mediated HR from dsDonor templates. Comparing networks of genes involved in HDR from different donor templates (ssDonor vs. plasmid dsDonor) revealed that many DNA repair factors are shared between SSTR and HR. The most striking commonality is the shared dependence on the FA pathway for both forms of repair. Despite this shared reliance on the FA pathway, we also observed genetic differences between the different types of HDR. Overall, this suggests that the early steps of HDR may be similar for templated repair of a Cas9 break with ssDonor or dsDonor, but the downstream stability and incorporation of different donor templates requires different factors. Future work could expand this platform to genome-wide screens or focus more narrowly on the FA pathway to determine if different FA sub-complexes act specifically during SSTR or HR.

By focusing on genes that restrict HDR and can also be targeted by small molecule inhibitors, we identified CDC7 inhibition as a strategy to boost HDR in human cells. We find that the small molecule inhibitor, XL413, occupies the sweet spot among HDR adjuvants of high efficacy, ease of use, and low toxicity. However, XL413 is ineffective in some cell types^29^. Thus, XL413-resistant cell lines could instead be treated with PHA-767491, if toxicity is not a limiting factor. Future development may yield CDC7 inhibitors that combine the consistency of PHA-767491 and the low toxicity of XL413.

As CDC7 plays a key role in DNA replication initiation, this fits well into the intimate link between DNA repair and cell cycle control^43^. CDC7 inhibition by XL413 both increases HDR and delays cells in S phase. The most parsimonious explanation is that XL413 promotes HDR by increasing the amount of time that cells spend in the HDR-permissive S phase. However, this model is partially at odds with observed stimulation of HDR with PHA-767491 treatment, which arrests cells in G1 phase prohibiting cells to move into S-phase^38^ (Supplementary Fig. 7e). This differential cell cycle profile between XL413 and PHA-767491 could potentially be explained by PHA-767491’s promiscuous activity against other kinases such as Cdk9, which is required for S-phase entry^44,45^. Another possibility is that CDC7 directly regulates DNA repair, which would be consistent with reports that CDC7-null yeast cells are sensitive to UV damage^46^. Mechanistic links between CDC7 and DNA repair remain unexplored, but might include direct phosphorylation of DNA repair proteins by CDC7. For example, CDC7 has been reported to phosphorylate the RAD18 ubiquitin ligase^47^, mutant alleles of which support increased HDR rates^48^. REV7 is also a target of CDC7 and forms a complex with shieldin to regulate DNA repair outcomes, and thus inhibition of CDC7 could have an indirect effect on DNA repair through REV7^49,50^. Another possible mode of interaction is scaffolding of DNA repair proteins by CDC7, which has been reported for RAD18^51^. Better understanding of the links between DNA replication control and DNA repair control may define these mechanisms and suggest more targeted interventions, for example preventing phosphorylation or degrading the key HDR-regulating substrate of CDC7.

The general link between HDR stimulation and cell cycle illustrates that HDR-boosting treatments can be a balancing act between desired and undesired outcomes. It is tempting to wish for a treatment that increases HDR to 100% efficiency, no matter the mechanism. The microtubule poison nocodazole boosts HDR in some contexts via G2/M arrest^7^, but also increases aneuploidy and failed mitoses (Fig. 6d, e). Similarly, inappropriately activating DNA repair during mitosis leads to chromosome fusions^52^. In our hands, CDC7 inhibition reversibly slows S-phase and effectively promotes stable genetic changes without increasing the rate of genomic instability. However, we cannot rule out that CDC7 inhibition has undesirable side-effects that remain to be discovered. Thus, CDC7 inhibition to increase HDR in the context of gene editing therapeutics warrants further investigation.

Here we demonstrate that understanding the genetics of gene editing reactions can be used to design interventions that favor certain types of DNA repair. While genetic CRISPR screens are a powerful tool to uncover phenotypes directly related to the expression levels of targeted genes, they fail to determine phenotypic outcomes that are caused by post-translational modifications. We anticipate that further work to map fundamental DNA repair pathways including studying the effects of post-translational changes of DNA repair proteins will suggest new strategies and targetable regulators to increase the precision and the efficacy of gene editing workflows.

## Methods

### Pooled screen

Replicate cultures of K562 cells stably expressing a dCas9-KRAB construct and a cassette containing a BFP reporter and a guide RNA targeting a library of DNA repair factors (Supplementary Data 1 GUIDES and previously described^9^) were thawed, cultured, and puromycin treated. 10e+06 cells from each replicate were subcultured (UNZAP). 25e+06 cells from each replicate were harvested for nucleofection. Each nucleofection aliquot was spun down, washed in PBS, and resuspended in 825 ml of nucleofection buffer SF (Lonza, Basel, Switzerland). Two hundred and seventy-five microliter of RNP editing mixture was added and mixed by pipetting. RNP for each replicate contained 2000 pmoles of sgRNA, 1,650 pmoles of Cas9, and 220 mg of plasmid pCR1075 donor DNA in Cas9 buffer (20 nM HEPES [pH 7.5]), 150 mM KCl, 1 mM MgCl_2_, 10% glycerol, and 1 mM TCEP). Each replicate of the RNP cell slurry was split and nucleofected in parallel in a Lonza 96-well Shuttle nucleofector (code FF-120), re-pooled, and cultured for two (replicate 1) or three (replicate 2) days. Nucleofected replicates were sorted into GFP + (GFP) and non-fluorescent (NON) populations on a Sony SH800S sorter. In parallel, an 85e+06 cell aliquot was harvested from each non-electroporated population the day of the sort (UNZAP), and an 85e+06 cell aliquot was harvested from each nucleofected cell library on the day of sorting (PRESORT). Harvested and sorted populations were spun down, rinsed in PBS, and frozen at −80 °C.

DNA from each cell population: PREZAP, UNZAP, PRESORT, GFP, and NON (non-treated) was purified using Machery-Nagel Blood Purification kits and the total amount of DNA quantified. One microgram of genomic DNA was amplified per Phusion HiFi PCR reaction using primers specific to the gRNA cassette as described^45^. Up to 24 PCR reactions were set up for each cell population to obtain desired coverage of the cell library. The thermocycler was set for one cycle of 98 °C for 30 s, 25 cycles of 98 °C for 15 s, 56 °C for 15 s, and 72 °C for 15 s, and one cycle of 72 °C for 10 min and held at 4 °C. PCR reactions were pooled, purified using SPRI beads, and sized on an agarose gel. Amplified DNA from each cell population was normalized to input cell numbers, purified a second time using SPRI beads, and sequenced on a HiSeq2500 (Illumina).

### Pooled screen analysis

Data analysis was performed as described^11^. Briefly, sequence reads were trimmed, aligned to DNA Repair Guide Sequences (Supplementary Data 1 GUIDES) and quantified. Read counts for each gRNA were normalized and compared to the distribution of untargeted control guides to determine significance and log2 magnitude of change. The top three guide-level phenotypes were collapsed to produce gene-level phenotype score. Results for the GFPvPRE comparison are available in (Supplementary Data 1).

### Pooled screen phenotype comparison

The magnitude of gene-level phenotype scores (calculated above) varied between screens. To facilitate direct comparison of essential genes between HR and SSTR results, essential genes (phenotype scores < 0) were unity normalized against all essential gene phenotype scores for the originating screen, such that *Z* = (phenotype_score − min(dataset))/(max(dataset)-min(dataset)). The resulting normalized values ranged from 0 (strongest phenotype score) to 1 (weakest phenotype score). *Z* values were binned for display purposes: bin 1, 0.0–0.2; bin 2, 0.2–0.4; bin 3, 0.4–0.6; bin 4, 0.6–0.8; and bin 5, 0.8–1.0. FA and related genes from either screen (FAAP20, UHRF1, TIP60, POLQ, and LIG4) with a phenotype score > 0 were assigned to bin 5. Raw data from this figure is presented in (Supplementary Data 1).

### Pooled screen GO-term comparison

Data from SSTR^9^ and HR (this manuscript) screens was filtered for statistical significance (*p* > 0.05) and separated into two categories: genes involved in SSTR or genes involved in HR. The gene list for each category was compared to the starting guide pool used in our screens (Supplementary Data 1 GUIDES) using DAVID v6.8^14^. Default search categories were used. Gene lists for GO categories are reported in (Supplementary Data 1 GO Terms).

### siRNA transfection

Approximately 1e+05 suspension or adherent cells were reverse transfected into 24-well plates using RNAiMAX (Thermo Fisher). siRNA (Supplementary Data 3) dosage was 40 nM. Cells were siRNA treated for 48 h, harvested, nucleofected, and recovered into media lacking siRNA. Verification of knockdown was performed at the time of nucleofection via qPCR. Cells were harvested for flow cytometry 4 days post nucleofection.

### Cell lines and culture

HEK293, HCT116, HeLa, U251, and K562 cells were acquired from the UC Berkeley Tissue Culture Facility. HEK293, HCT116, and HeLa cells were maintained in DMEM medium supplemented with 10% fetal bovine serum and Penicillin/Streptomycin. K562 cells were maintained in RPMI medium supplemented with 10% fetal bovine serum and Penicillin/Streptomycin. U251 cells were maintained in DMEM-F12 medium supplemented with 10% fetal bovine serum and Penicillin/Streptomycin. Cell lines were tested regularly for mycoplasma contamination using enzymatic (Lonza, Basel, Switzerland) and PCR-based assays (Bulldog Bio, Portsmouth, New Hampshire). Primary mouse glial cells were extracted as described^53^ and maintained in DMEM medium supplemented with only 10% fetal bovine serum. Human dermal lymphatic endothelial cells (HDLECs) were maintained in EBM-2 MV media from Lonza. Human iPSCs were generated from dermal fibroblasts from a healthy male subject (WTc) using the episomal reprogramming method^54^. iPSCs were cultured in Stemfit (Ajinomoto) on matrigel coated plates at 37 °C. After passaging, iPSCs were plated in Stemfit with Rock inhibitor Y-27632 (SelleckChem). Human dermal fibroblasts (HDFs) were derived from a healthy male subject and cultured in DMEM supplemented with 1% sodium pyruvate (Thermo Fisher), 10% fetal bovine serum and Penicillin/Streptomycin. Derivation and use of human iPSCs and HDFs was approved by the UCSF Committee on Human Research, San Francisco, CA (IRB 10-02521). All subjects provided written informed consent prior to participation. Cryopreserved wildtype human mobilized peripheral blood CD34^+^ HSPCs from volunteer donors were purchased from Allcells, and were cultured in SFEMII + CC110 (StemCell Technologies).

### T Cell isolation and culture

Primary human T cells were isolated from two de-identified healthy human donors from residuals from leukoreduction chambers after Trima Apheresis (Vitalant), with written informed consent under a protocol approved by the UCSF IRB (BU101283). Peripheral blood mononuclear cells (PBMCs) were isolated by Ficoll centrifugation using SepMate tubes (STEMCELL, per manufacturer’s instructions), then T cells were further isolated from PBMCs by magnetic negative selection using an EasySep Human T Cell Isolation Kit (STEMCELL, per manufacturer’s instructions). Isolated T cells were cultured at 1 million cells mL^−1^ in XVivo15 medium (STEMCELL) with 5% fetal bovine serum, 50 μM 2-mercaptoethanol, and 10 mM N-Acetyl l-Cystine, and stimulated for 2 days prior to electroporation with anti-human CD3/CD28 magnetic dynabeads (ThermoFisher) at a beads to cells concentration of 1:1, along with a cytokine cocktail of IL-2 at 200 U mL^−1^ (UCSF Pharmacy), IL-7 at 5 ng mL^−1^ (ThermoFisher), and IL-15 at 5 ng mL^−1^ (Life Tech). T cells were harvested from their culture vessels and magnetic dynabeads were removed by placing cells on an EasySep cell separation magnet for 5 min. Immediately prior to electroporation, de-beaded cells were centrifuged for 10 min at 90 g, media aspirated, and resuspended in the Lonza electroporation buffer P3 using 20 µL buffer per one million cells.

### Cas9, RNA, and donor DNA preparation

Streptococcus pyogenes Cas9 (pMJ915, Addgene #69090) with two nuclear localization signal peptides and an HA tag at the C-terminus was expressed in Rosetta2 DE3 (UC Berkeley Marcolab) cells. Cell pellets were sonicated, clarified, Ni2+ -affinity purified (HisTraps, GE life sciences), TEV cleaved, cation-exhanged (HiTrap SP HP, GE life sciences), size excluded (Sephacryl S-200, GE life sciences) and eluted at 40 in 20 mM HEPES KOH pH 7.5, 5% glycerol, 150 mM KCl, 1 mM dithiothreitol (DTT)^55^. Alternatively, Streptococcus pyogenes Cas9-NLS was obtained from the QB3 MacroLab at UC Berkeley.

sgRNAs were synthesized by Synthego as modified gRNAs with 2′-O-methyl analogs and 3′ phosphorothioate internucleotide linkages at the first three 5′ and 3′ terminal RNA residues using protospacer sequences described in (Supplementary Data 3).

crRNAs/tracrRNAs were chemically synthesized (Edit-R, Dharmacon Horizon) using protospacer sequences described in (Supplementary Data 3).

ssDonors were obtained by ordering unmodified Ultramer oligonucleotides (Integrated DNA Technologies).

dsDonor was obtained by purifying plasmid DNA from bacterial cultures containing the indicated plasmid (Qiagen) or by SPRI purification of long double-stranded PCR amplicons.

### Plasmid constructs

Sequences for plasmids used in this study are described in (Supplementary Data 3). AICSDP-8:TOMM20-mEGFP, AICSDP-19:LAMP1-mEGFP, AICSDP-13:FBL-mEGFP, AICSDP-50: NPM1-mEGFP, AICSDP-57: SMC1A-mEGFP, AICSDP-64: FUS-mEGFP, AICSDP-52: HIST1H2BJ-mEGFP were gifts from The Allen Institute for Cell Science (Addgene plasmid #s 87423, 101782, 87427, 109122, 114406, 114410, and 109121). pCR1063, pCR1068, and BFP dest clone were gifts from Jacob Corn (Addgene plasmid #s 111091, 111092, 71825).

### Cas9 RNP assembly and nucleofection

Fifty pmoles of sgRNA was diluted using Cas9 buffer (20 nM HEPES [pH 7.5]), 150 mM KCl, 1 mM MgCl_2_, 10% glycerol, and 1 mM TCEP) or water. 1.25 μL of 40 mM Cas9-2xNLS (50 pmoles) was slowly mixed in, and the resulting mixture was incubated for 5 min at room temperature to allow for RNP formation. After incubation, either 0.5 μL of 100 μM ssDonor or 1.5 or 2 μg of plasmid DNA was introduced and mixed by pipetting. The total volume of RNP solution was 5 ml, where the volume of Cas9 buffer was adjusted to account for volume differences between ssDonor and plasmid DNA. Between 1e+05 and 2e+05 cells were harvested, washed once in PBS, and resuspended in 15 μL of nucleofection buffer (Lonza, Basel, Switzerland). Five microliter of RNP mixture was added to 15 μL of cell suspensions. Reaction mixtures were electroporated in Lonza 4D nucleocuvettes, incubated in the nucleocuvette at room temperature for five minutes, and transferred to culture dishes containing pre-warmed media. Large-scale nucleofections were performed by splitting cultures and conducting multiple parallel nucleofections.

Editing outcomes were measured four days post-nucleofection by flow cytometry or by amplicon sequencing (see below). Resuspension buffer and electroporation conditions were the following from each cell line: K562 in buffer SF with FF-120, HEK293 in buffer SF with DS-150, T cells in buffer P3 with EH-115, primary mouse glial cells in buffer P3 with DS-112, HDLECs in buffer P3 with CA-137 HCT116s in buffer SE with EN-113, and HeLa cells in buffer SE with CN-114, U251 cells in buffer SE with DS130, iPSCs in buffer P3 with DS-138, HDFs in buffer P3 with DS-150 and HSPCs in buffer P3 with ER-100.

Unless otherwise indicated, cells were separated into three stocks, then nucleofected, recovered, and analyzed separately (biological triplicate).

### T Cell nucleofections

RNPs were generated immediately prior to electroporation. Briefly, crRNA targeting *RAB11A*, *TUBA1B*, *CLTA*, exon 6 of CD4, or five genomic loci (Chr5GD1, Chr5GD2, CD55_1, CD55_2, and CD59_2) and tracrRNAs were chemically synthesized (Edit-R, Dharmacon Horizon), and Cas9-NLS was recombinantly produced and purified (QB3 Macrolab). Lyophilized RNA was resuspended in 10 mM Tris-HCL (7.4 pH) with 150 mM KCl at a concentration of 160 µM, and stored in aliquots at −80 °C. crRNA and tracrRNA aliquots were thawed, mixed 1:1 by volume, and annealed by incubation at 37 °C for 30 min to form an 80 µM gRNA solution. This was further mixed 1:1 by volume with 40 µM Cas9-NLS protein to achieve a 2:1 molar ratio of gRNA:Cas9, with final RNP concentration of 20 µM. Long linear dsDonor templates creating an N-terminal fusion protein of a fluorescent protein (GFP, BFP or mCherry) and the Rab11a, Tubulin, or Clathrin Light Chain A proteins^22^ were generated as a PCR amplicon using KapaHiFi polymerase (Kapa Biosystems), purified by SPRI bead cleanup, and resuspended in water to 125 ng µL^−1^ as measured by light absorbance on a NanoDrop spectrophotometer (Thermo Fisher). For CD4 SNP introduction, ssDonor templates were synthesized by IDT Technologies encoding the dbSNP: rs28919570 in-frame missense mutation (NM_000616.5c.793C>T, p.Arg265Trp). All ssDonor sequences are listed in (Supplementary Data 3).

Fifty picomolar of RNP and 0.25 μg of dsDonor, or 100 pmol of ssDonor, were mixed for 5–10 min, then added to cells 3–5 min before electroporation. One million T cells per well with RNP and donor template were electroporated using the Lonza 4D 96-well electroporation system with pulse code EH115, in biological replicate of *n* = 2–3. Immediately post-electroporation, prewarmed media was added to rescue the cells then each electroporation condition was split into 5 wells of a 96-well U-bottom tissue culture plate. Electroporated cells were incubated at 1 million cells mL^−1^ in final volume 200 μL media with IL-2 at 500 U mL^−1^ and increasing concentrations of XL413. After 24 h, all cells were washed in PBS, and fresh media was added containing only IL-2 at 500 U mL^−1^. Approximately 3 days post-electroporation, cells were collected by centrifugation at 300 × *g*, media discarded, and antibody stains were added (UCHT1-CD3-PE, OKT4-CD4-PE-Cy7, RPA-T8-APC (all from BioLegend), and GhostDye780 (Tonbo)) for 20 min (Supplementary Data 3). To assess conversion to the CD4^SNP^ sequence, SK3-CD4-FITC (Biolegend) was also added to the antibody staining. Cells were washed, resuspended in PBS + 1% serum (120 μL per well), and then an equal volume (80 μL) of each well was sampled using an Attune NxT Focusing Flow Cytometer with Autosampler attachment (ThermoFisher).

### Genomic DNA extraction (for amplicon sequencing)

Approximately 1e+05 cells were harvested and resuspended in 50 μL of QuickExtract DNA extract solution (Lucigen). Reactions were incubated for 20 min at 65 °C and 5 min at 95 °C. Extractions were diluted 1:4 with dH2O, spun for 5 min at max speed in a microcentrifuge, and the supernatants retained for downstream analysis.

### PCR amplification of edited regions

PCR reactions were generated from 2× Q5 master mix (NEB), primers (Supplementary Data 3) at 500 nM, and 5 μL of genomic DNA (see above). Unless otherwise noted, PCR primers have a 5′ sequence tag (GCTCTTCCGATCT) that allows re-amplification for Illumina sequencing (amplify-on PCR). The thermocycler was set for one cycle of 98 °C for 1 min, 35 cycles of 98 °C for 10 s, 63 °C for 15 s, 72 °C for 60 s, and one cycle of 72 °C for 4 min, and held at 4 °C. PCR amplicons were purified using SPRI beads, run on a 1.5% agarose gel to verify size and purity, and quantified by Qubit (Thermo Fisher, Waltham, MA).

### NGS library generation and sequencing

Illumina adapters and index sequences were added to 100 ng of purified PCR amplicons by amplify-on PCR. Amplify-on was performed using 100 ng of template DNA, 0.5 μM of forward/reverse primers, and 2× Q5 Master Mix (NEB). The thermocycler was set for one cycle of 98 °C for 30 s, 16 cycles of 98 °C for 10 s, 62 °C for 20 s, 72 °C for 30 s, and one cycle of 72 °C for 1 min, and held at 4 °C. Each adapter-conjugated amplicon was quantified by qubit, normalized, and pooled at equimolar amounts. Pooled samples were purified using SPRI beads. Library size and purity was verified by Bioanalyzer trace prior to sequencing on an Illumina MiSeq using reagent kit v3 (2 × 300 bp).

### NGS analysis of amplicons

Samples were deep sequenced on an Illumina MiSeq at 300 bp paired-end reads to a depth of at least 10,000 reads per sample. Cortado (https://github.com/staciawyman/cortado v1.0) was used to analyze editing outcomes. Briefly, reads were adapter trimmed then joined before performing a global alignment between reads and the reference and donor sequences using NEEDLE^56^. Rates of HDR are calculated as total number of reads that are successfully converted to the donor sequence (and have no insertions or deletions at the cut site) divided by the total number of aligned reads. NHEJ rates are calculated as any reads where an insertion or deletion overlaps the cut site or occurs within a six base pair window around the cut site divided by the total number of aligned reads. SSTR/HDR rates were calculated at specific regions by counting total number of reads with flag occurrence divided by the number of aligned reads. Amplicon sequencing results can be found in (Supplementary Data 2).

### Compound treatment

Acyriaflavin A, XL413, Aphidicolin, GW843682X, L755507, PHA-767491, and Ro3280 were sourced from Tocris. Hydroxyurea, A64 trifluoroacetate, GKT136901, SB220025, nocodazole, and UNC2170 trifluoroacetate were sourced from Sigma.

Approximately 1e+05 suspension or adherent cells were seeded post-nucleofection into 96-well plates in media supplemented with the following concentrations of compounds: Acyriaflavin A at 5 μM, XL413 at 33 μM, Hydroxyurea at 2 μM, SRC7 at 1 μM, RS1 at 10 μM, Aphidicolin at 2 mg mL^−1^, A64 trifluoroacetate at 1 μM, SB220025 at 0.5 μM, GKT136901 at 50 μM, GW843682X at 0.5 μM, Ro3280 at 100 nM, UNC2170 trifluoroacetate at 150 μM, nocodazole at 33 ng μL^−1^, and L755507 at 5 μM. Cells were compound treated for 24 h, harvested, washed, and recovered into fresh media. Cells were harvested for flow cytometry or amplicon sequencing 4 days post nucleofection. Nocodazole treatments were administered as compound-treated media 24 h pre-nucleofection instead of post-nucleofection.

i53 treated cells were transfected using Lipofectamine 2000 from Invitrogen. Three microgram of each of three plasmids containing a broken i53 gene (Addgene #94939), an intact i53 gene (Addgene #74940), and both an intact i53 gene and a BFP reporter (Addgene #111145, respectively, were incubated in OPTI-MEM with Lipofectamine 2000 for 30 min. The cells were then incubated with the Lipofectamine plasmid solution for 24 h, pre-nucleofection. The BFP reporter was used to analyze the transfection efficiency of the i53 plasmid into the cells by flow cytometry.

### qPCR

Between 1e+05 and 2e+05 cells were harvested and RNA extracted using Qiagen RNeasy kits (Qiagen, Venlo, Netherlands). cDNA was produced from 1 mg of purified RNA using the iScript Reverse Transcription Supermix (Bio-Rad). qPCR reactions were performed using the SsoAdvanced Universal SYBR Green Supermix (Bio-Rad) in a total volume of 10 mL with primers at final concentrations of 500 nM. The thermocycler was set for 95 °C for 2 min and 40 cycles of 95 °C for 2 s and 55 °C for 8 s. Fold enrichment of the assayed genes over the control *ACT1B* and/or *GAPDH* loci were calculated using the 2^−ΔΔCt^ method essentially as described^57^. Primer sequences can be found in Supplementary Data 3.

### Western blotting

Cells were harvested and washed with PBS. Cell were lysed in 1× RIPA buffer (EMD Millipore) for 10 min on ice. Samples were spun down at 14,000 × *g* for 15 min, and cleared protein lysates were transferred to a new tube. Twenty milligram of RIPA protein lysate was resolved on a TGX anyKD gel (Bio-Rad) and semi-dry transferred (TransBlot Turbo, Bio-Rad) to nitrocellulose membranes. Membranes were blocked in 5% milk, incubated with primary antibodies in 5% milk, incubated with secondary antibodies in 5% milk, and exposed on a LiCor Odyssey CLx (Li-Cor). Antibodies used were: FLAG (Sigma F1804, 1:1000), GAPDH (cell Signaling 97166, 1:5000), phospho-S53 MCM2 (Abcam ab109133, 1:1000), MCM2 (Abcam ab6153, 1:1000), 1:10,000 donkey anti-mouse IgG-IR800 (Li-Cor 925-32212), 1:10,000 donkey anti-mouse IgG-IR680 (Li-Cor 925-68022), 1:10,000 donkey anti-rabbit IgG-IR800 (Li-Cor 925-32213), 1:10,000 donkey anti-rabbit IgG-IR680 (Li-Cor 925-68023).

### Biallelic editing assays

To check for frequencies of multi-allelic knock-ins in K562 cells, cells were nucleofected with RNPs targeting *FUS*, *HIST1H2BJ* or *SMC1A* and corresponding dsDonors, respectively, and were kept in complete media or media treated with 10 μM XL413 for 24 h. After 24 h the drug was removed and cells cultured for an additional 3 days. Four days of post nucleofection cells were analyzed for GFP fluorescence using an Aria II (BD Biosciences) cell sorter. GFP positive cells in both treated and untreated samples were sorted as single cells into 96-well culture plates containing complete media. Single-cell clones were grown for 10 days and DNA was extracted using a QuickExtract DNA extraction buffer (Lucigen). PCR across the C-terminal insertion site was performed using Q5 polymerase (New England Biolabs). Primers for PCRs can be found in Supplementary Data 3. PCR products were run on a 1% agarose gel and genotypes were determined. Clones classified as heterozygotes showed both the wild-type and knockin PCR band and clones classified as homozygotes only showed the knock-in PCR band (higher molecular weight). Clones that only showed the wildtype band are named no knock-in but could be considered as clones that show integration of the GFP sequence somewhere else in the genome (as they were in the GFP positive cell pool but do not show integration at the target site). In some clones we observed deletional bands. These clones were excluded in our analysis.

To quantify biallelic editing rates in T cells, cells were nucleofected with RNPs targeting the *RAB11A* locus as described above but with two different dsDonor constructs in a 1:1 ratio, one of which contains a BFP reporter sequence and one of which contains an mCherry reporter. Cells were treated with increasing amounts (0, 3.3, 10, and 33 μM) of XL413. After 4 days cells were analyzed for successful knock-in by flow cytometry and frequencies of double positive cell populations (BFP^+^ and mCherry^+^) were determined that represent the biallelic knock-in rates.

### FUCCI cell line generation

K562 cells were electroporated with 40 µg pML143 donor vector containing EF1α-mAG1-geminin(1–110)-P2A-mKO2-hCdt1(30–120) flanked by homology arms to AAVS1/PPP1R12C locus and 5 µg each of AAVS1L and AAVS1R TALENs. Targeted cells were sorted by FACS into 96-well plates to isolate single clones. Clonal cell lines were assayed by live cell imaging and flow cytometry to verify correct correlation with fluorescence and cell cycle progression.

### Cell cycle assay/DNA quantification assay

K562 FUCCI cells were grown in complete RPMI containing Aphidicolin (2 mg mL^−1^), Hydroxyurea (2 mM) or XL413 (33 μM). Cells were harvested at indicated time points and subjected to flow cytometry. Cell cycle status was determined gating for mAG1^+^/mKO2^−^(S/G2/M), mKO2^+^/ mAG1^−^ (G1), mAG1^+^/ mKO2^+^ (early S) and mAG1^−^/mKO2^−^ (early G1) cell populations. DNA content of the cells was determined using Hoechst33342 (Thermo Fisher) DNA dye. Hoechst33342 was added to cells in culture medium (final concentration 1 mg mL^−1^) for 30 min at 37 °C before cells were subjected to flow cytometry.

Cell cycle analysis was also performed by PI staining with flow cytometry. Cells were incubated in 0.5 % Triton X-100 in PBS, treated with RNase A, stained with 5 μL propidium iodide (1 mg mL^−1^) and analyzed by flow cytometry.

### Addgene constructs

A full list of reagents used in this manuscript can be found in Supplemental Data 3. BFP dest clone was a gift from Jacob Corn (Addgene plasmid # 71825; http://n2t.net/addgene:71825; RRID:Addgene_71825). AICSDP-19:LAMP1-mEGFP was a gift from The Allen Institute for Cell Science (Addgene plasmid # 101782; http://n2t.net/addgene:101782; RRID:Addgene_101782). AICSDP-13:FBL-mEGFP was a gift from The Allen Institute for Cell Science (Addgene plasmid # 87427; http://n2t.net/addgene:87427; RRID:Addgene_87427). AICSDP-8:TOMM20-mEGFP was a gift from The Allen Institute for Cell Science (Addgene plasmid # 87423; http://n2t.net/addgene:87423; RRID:Addgene_87423). AICSDP-50: NPM1-mEGFP was a gift from The Allen Institute for Cell Science (Addgene plasmid # 109122; http://n2t.net/addgene:109122; RRID:Addgene_109122). AICSDP-57: SMC1A-mEGFP was a gift from The Allen Institute for Cell Science (Addgene plasmid # 114406; http://n2t.net/addgene:114406; RRID:Addgene_114406). AICSDP-64: FUS-mEGFP was a gift from The Allen Institute for Cell Science (Addgene plasmid # 114410; http://n2t.net/addgene:114410; RRID:Addgene_114410). AICSDP-52: HIST1H2BJ-mEGFP was a gift from The Allen Institute for Cell Science (Addgene plasmid # 109121; http://n2t.net/addgene:109121; RRID:Addgene_109121).

### Reporting summary

Further information on research design is available in the Nature Research Reporting Summary linked to this article.

## Supplementary information

Supplementary Dataset 1

Supplementary Dataset 2

Supplementary Dataset 3

Supplementary Information

Reporting Summary

Description of Additional Supplementary Files

## Data Availability

Sequencing reads used in the pooled screen and amplicon sequencing data were deposited in SRA as PRJNA610420. Plasmid constructs are described in Supplementary Data 3. All other relevant data are available from the authors upon reasonable request.

## Source Data

Source Data

## Code availability

Code used for amplicon sequencing data processing is available at https://github.com/staciawyman/cortado.
