# Supplementary material for: Timed inhibition of CDC7 increases CRISPR-Cas9 mediated templated repair
Source: Nat Commun. 2020 Apr 30;11:2109. doi: 10.1038/s41467-020-15845-1 (PMC7193628; doi:10.1038/s41467-020-15845-1)
Supplement: Supplementary file 4 — Supplementary Information [file 41467_2020_15845_MOESM4_ESM.pdf]

Supplementary information for the article

Timed inhibition of CDC7 increases CRISPR-Cas9 mediated templated repair

Beeke Wienert[1,2,3]*; David N Nguyen[4,5,6]*; Alexis Guenther[7]; Sharon J Feng[1,2]; Melissa N Locke[2]; Stacia K Wyman[1]; Jiyung Shin[8]; Katelynn R Kazane[1,2]; Georgia L Gregory[3]; Matthew AM Carter[3]; Francis Wright[4], Bruce R Conklin[3,9], Alex Marson[1,4,5,6,10,11], Chris D Richardson[1,2,7] ‡; Jacob E Corn[1,2,8] ‡

[1]Innovative Genomics Institute, University of California, Berkeley, CA, 94703

[2]Department of Molecular and Cell Biology, University of California, Berkeley, CA, 94703

[3]Gladstone Institutes, San Francisco, CA, 94158

[4]Department of Microbiology and Immunology, University of California, San Francisco, CA, 94143

[5]Diabetes Center, University of California, San Francisco, San Francisco, CA, 94143

[6]Department of Medicine, University of California, San Francisco, CA, 94143

[7]Department of Molecular, Cellular, and Developmental Biology, University of California, Santa Barbara, CA, 93106

[8]Institute of Molecular Health Sciences, ETH Zürich, 8093 Zurich, Switzerland

[9]Departments of Medicine, Ophthalmology, and Pharmacology, University of California, San Francisco, CA, 94143

[10]Parker Institute for Cancer Immunotherapy, San Francisco, CA, 94129

[11]Chan Zuckerberg Biohub, San Francisco, CA, 94158

*Authors contributed equally to this work

‡Co-corresponding. Correspondence: jacob.corn@biol.ethz.ch, chris.richardson@lifesci.ucsb.edu

Supplementary Figure 1

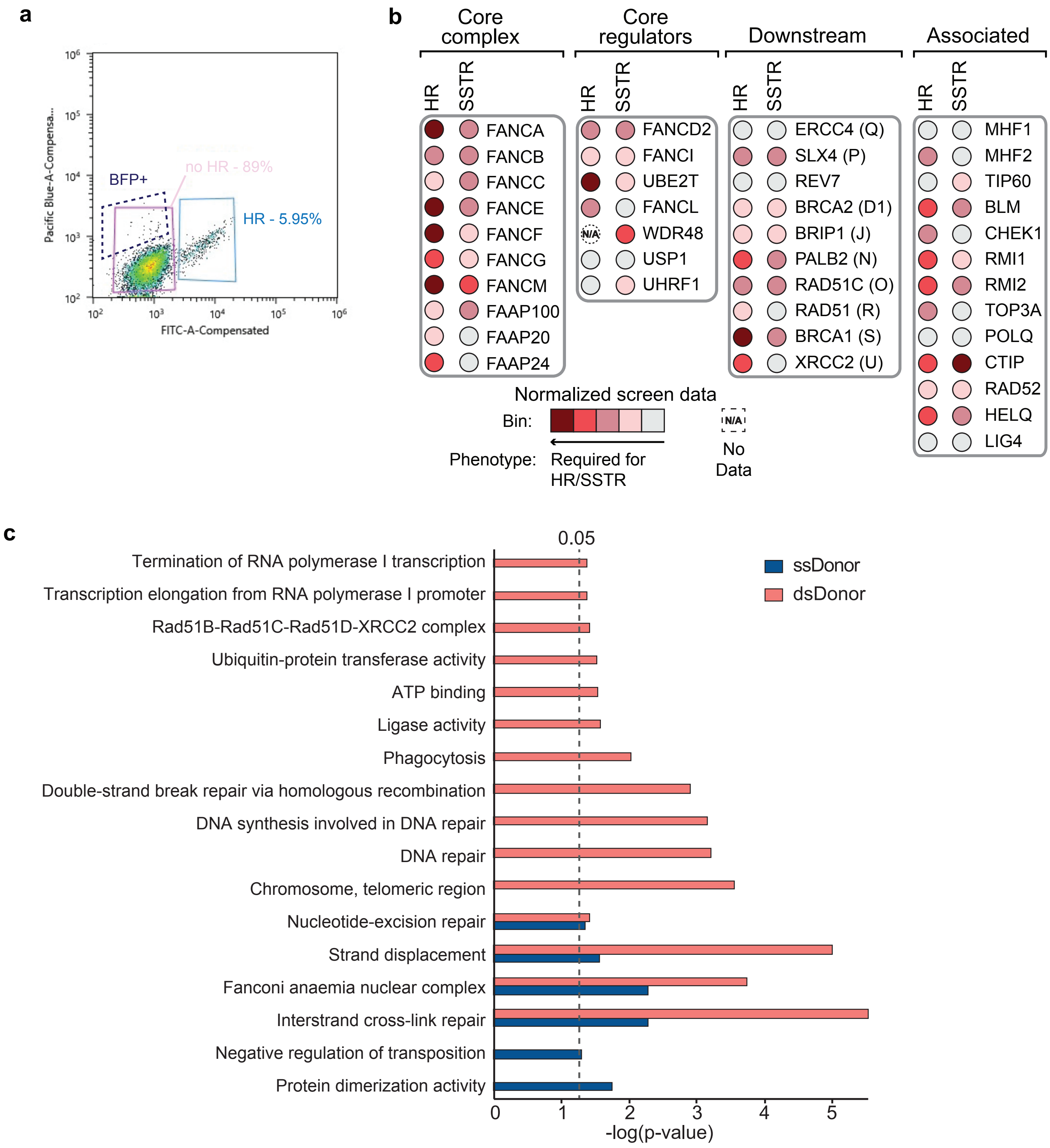

d

**Drug**	**Target**	**Gene Function**
Acyriaflavin A	CCND1	Regulatory subunit of CDK4 or CDK6, required for cell cycle G1/S transition.
XL413	CDC7	Kinase. Critical for the G1/S transition.
A64 trifluoroacetate	HIPK2	Conserved serine/threonine kinase.
SB220025	MAPK14	Kinase. Functions in cell cycle regulation.
GKT136901	NOX4	Catalytic subunit the NADPH oxidase complex.
GW843682X	PLK3	Kinase. Regulator of cell cycle progression.
Ro3280	PLK1	Kinase.Early trigger for G2/M transition.
UNC2170 trifluoroacetate	TP53BP1	DSB protein. Promotes NHEJ, and limits homologous recombination.

Supplementary Figure 1: A pooled CRISPR screen reveals regulators of HR. **(a)** Example data from pooled screen sort showing gates used to isolate non-fluorescent and GFP^+ cell populations. Location of BFP+ (unedited or perfectly repaired) cells highlighted with a dashed box. Data shown is for replicate 1. **(b)** The FA repair pathway is required for HR and SSTR. Gene names are annotated with normalized phenotype scores (see **Methods**) for both HR and SSTR screens. Increasing color intensity and decreasing bin number is proportional to the effect size seen in the HR or SSTR screen data. Functional FA complexes: the FA Core complex, Core regulators influencing FANCD2-FANCI ubiquitination, Downstream repair effectors, and Associated factors. Raw data presented in **[Supplementary Table 1]**. **(c)** Unique and shared genetic pathways contribute to SSTR and HR. GO term analysis for statistically significant ($p<0.05$, modified Fisher exact p-value) hits from HR and SSTR screens. Data presented as GO terms enriched in HR dataset (dsDonor, orange) or SSTR dataset (ssDonor, blue). Bar plot heights present statistical significance of GO term enrichment. Raw data presented in **[Supplementary Table 1]** or prior publication[9]. Data represent two biological replicates each of the HR and SSTR screens. **(d)** Factors restricting SSTR and HR can themselves be inhibited by small molecules. HDR repressors identified from our screens **[Fig. 1],** which can be targeted with known small molecule inhibitors, are listed with the small molecule inhibitors tested in **[Fig. 2]**, the inhibitor-targeted genes, and the gene function.

Supplemetary Figure 2

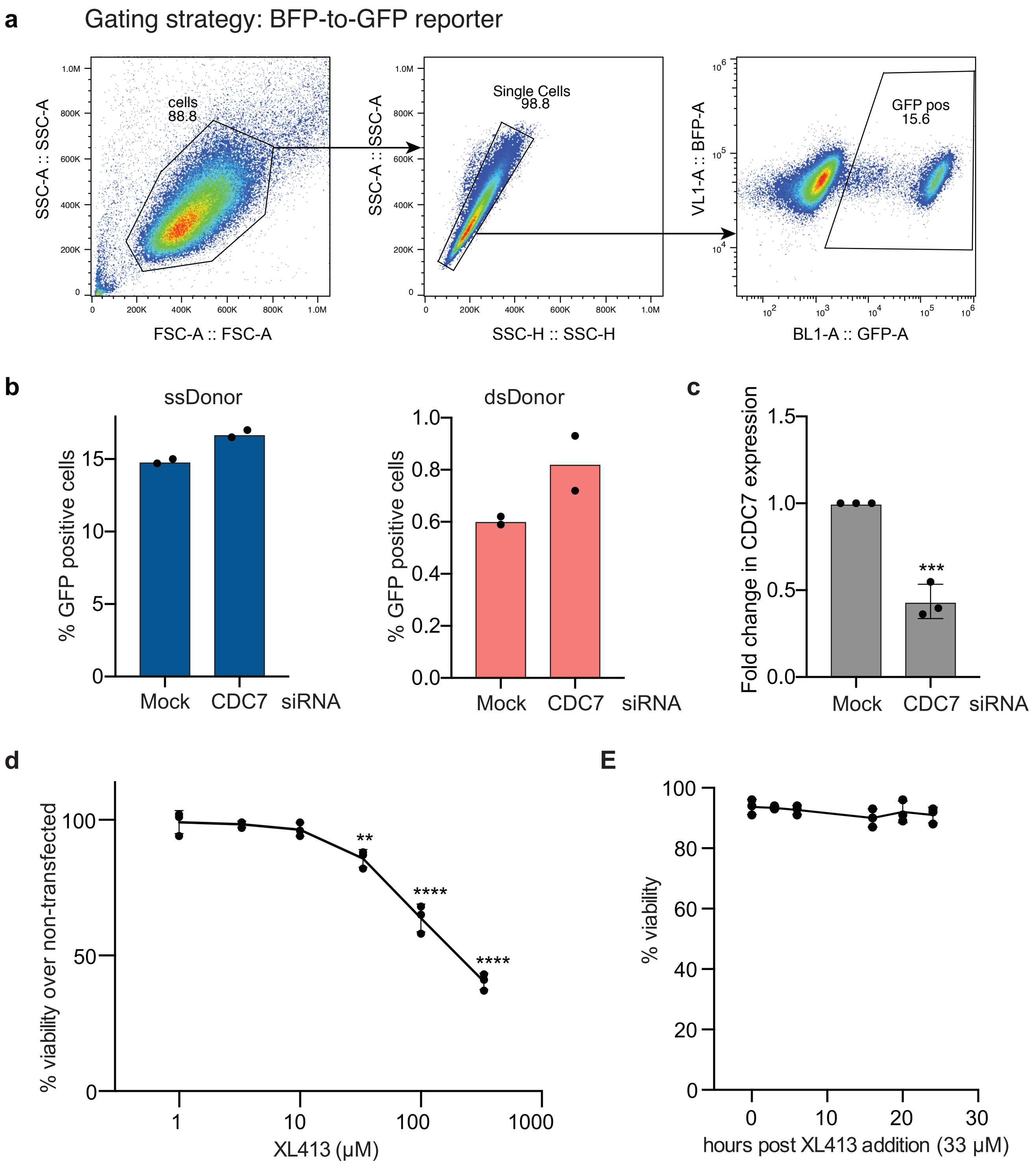

Supplementary Figure 2: Chemical or transcriptional inhibition of CDC7 promotes templated repair events. (**a**) Gating strategy to determine BFP-to-GFP conversion rates in panels (B), (D-E), Fig. 1 and Fig. 2. Cells were gated for size by FSC and SSC (left), then single cells were selected, then GFP and BFP fluorescence was determined. **(b)** siRNA inhibition of CDC7 influences SSTR and HR. K562 cells treated with siRNA against mock sequence or CDC7 were edited with the indicated RNP and donor DNA and %GFP was plotted for each population as analyzed by flow cytometry. Data presented as mean±SD (n=2). **(c)** Fold depletion of the target transcript normalized to controls (*ACTB, GAPDH*) was measured by qPCR. Data presented were calculated from n=3 cell pellets harvested at the time of electroporation. **(d)** Increasing doses of XL413 can be toxic in K562-BFP cells. Viability after nucleofection and treatment with XL413 at indicated concentrations for 24h was determined after 4 days by flow cytometry by measuring events in characteristic forward and side scatter gates; viability was normalized to non-transfected K562 cells. (**e**) Working dose of XL413 is well tolerated. Viability of K562 cells at different time points post XL413 addition (33 μM). Viability was determined using Trypan blue exclusion test. All values are shown as mean±SD (n=3 biological replicates). Statistical significances were calculated by unpaired two-tailed t-test using the Holm-Sidak method (b and c) or ordinary one-way ANOVA and Dunnet's multiple comparison test (d and e) (p-values are reported as *p<0.05, **p<0.01, ***p<0.001, ****p<0.0001).

Supplementary Figure 3

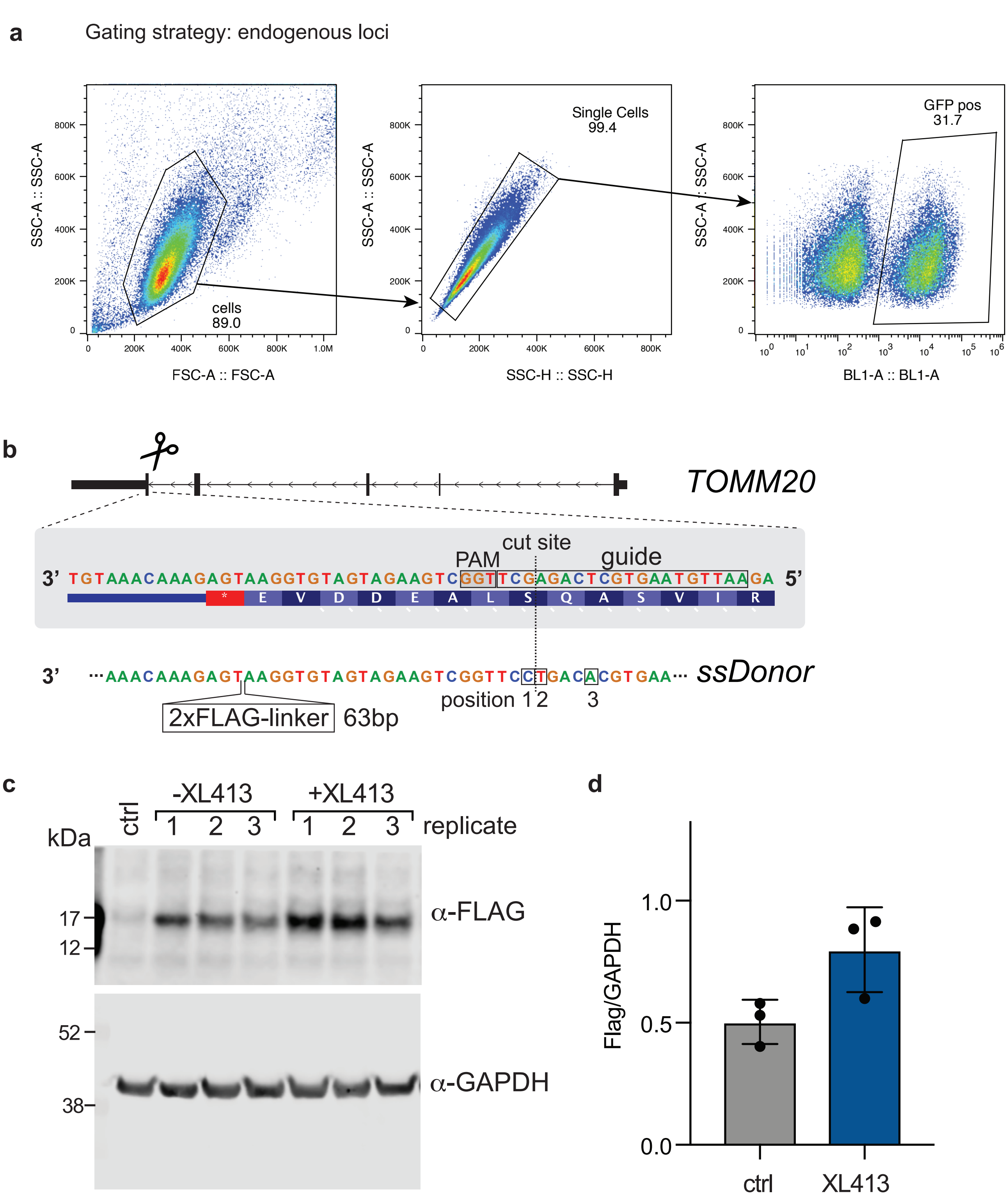

Supplementary Figure 3: Editing and flow cytometry strategies. (**a**) Gating strategy to determine endogenous GFP expression after genome editing in Figs. 3a, 6a-d and Supplementary Figs. 6b-c, 7a-b, 8, and 9h. Cells were gated for size by FSC and SSC (left), then single cells were selected, then GFP fluorescence was determined. (**b**) Schematic outlining the genome editing strategy to knock-in a 2xFLAG sequence at the C-terminus of TOMM20 using a ssDonor. A linker (GGGGS)-2xFLAG sequence (63 bp) and three additional synonymous point mutations (to prevent repeated cutting) are introduced near the Cas9 target site. **(c)** XL413 promotes on-target editing. Western blot analysis for FLAG expression in non-transfected K562 cells (ctrl) and nucleofected K562s cells with and without treatment with XL413. Cells were nucleofected with RNPs targeting *TOMM20* and 2xFLAG ssDonor in the presence or absence of 10 μM XL413 for 24 h. Cells were harvested for protein extraction 4 days post nucleofection. Data presented is representative of Western blots on n=2 experiments. **(d)** Quantification of Western blot band intensities in (d) by gel densitometry. Values are shown as mean±SD (n=3 biological replicates). Statistical significances were calculated by unpaired two-tailed t-test using the Holm-Sidak method.

Supplementary Figure 4

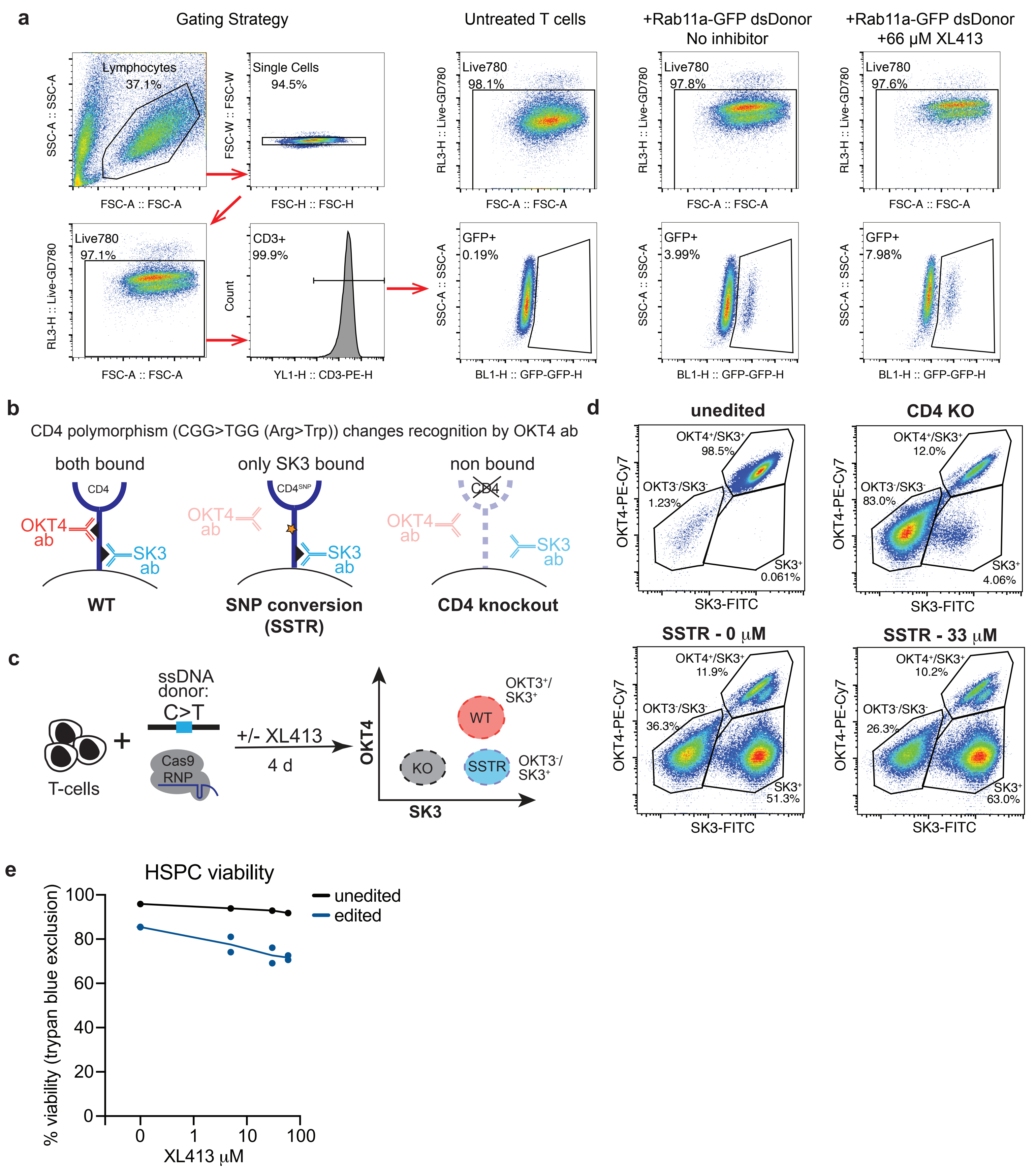

Supplementary Figure 4: Effect of XL413 inhibitor on growth of primary human T cells. **(a)** Panels from representative samples showing flow cytometry plots depicting gating strategy, viability (top) and GFP positivity (bottom) from electroporated $CD3^+$ T cells at day 3 that are either untreated or nucleofected with RNP and RAB11A-GFP donor +/- XL413. $CD3^+$ T-cells from two healthy donors were nucleofected with RNPs targeting *RAB11A* and a linear dsDonor template that encodes an N-terminal fusion of *GFP* to the *RAB11A* gene. XL413 (66 μM) was added to the growth media for 24 h post-editing. Viability was determined by staining with GhostDye780. Panels from representative samples showing flow cytometry plots depicting viability (top) and GFP positivity (bottom) from electroporated $CD3^+$ T cells at day 3 that are either untreated or nucleofected with RNP and RAB11A-GFP donor +/- XL413. **(b)** Schematic of CD4 SNP conversion assay. Cells with wildtype CD4 on the cell surface are recognized by both OKT4 and SK3 monoclonal antibodies at different epitopes. Mutation of the OKT4 epitope by SSTR results in an in-frame missense SNP ($CD4^{SNP}$, rs28919570, p.Arg265Trp) that binds only SK3 but not OKT4. Disruption of CD4 by frame-shift mutation or ablation eliminates both OKT4 and SK3 binding. **(c)** $CD4^{SNP}$ editing workflow. Cas9-RNPs targeting the CD4 locus are nucleofected into $CD3^+$ T cells along with ssDonor encoding the R265W amino acid change. WT, SSTR, and KO populations can be resolved by flow cytometry. **(d)** Representative flow cytometry plots, with same initial gating strategy as in (a), for an unedited (top left) and ssDonor edited T cell populations showing that the assay can discriminate successful SSTR from WT or KO scenarios. **(e)** XL413 and nucleofection both slightly reduce viability of primary human HSPCs. Viability was determined by Trypan blue exclusion test.

All values are shown as mean±SD (biological replicates as indicated).

Supplementary Figure 5

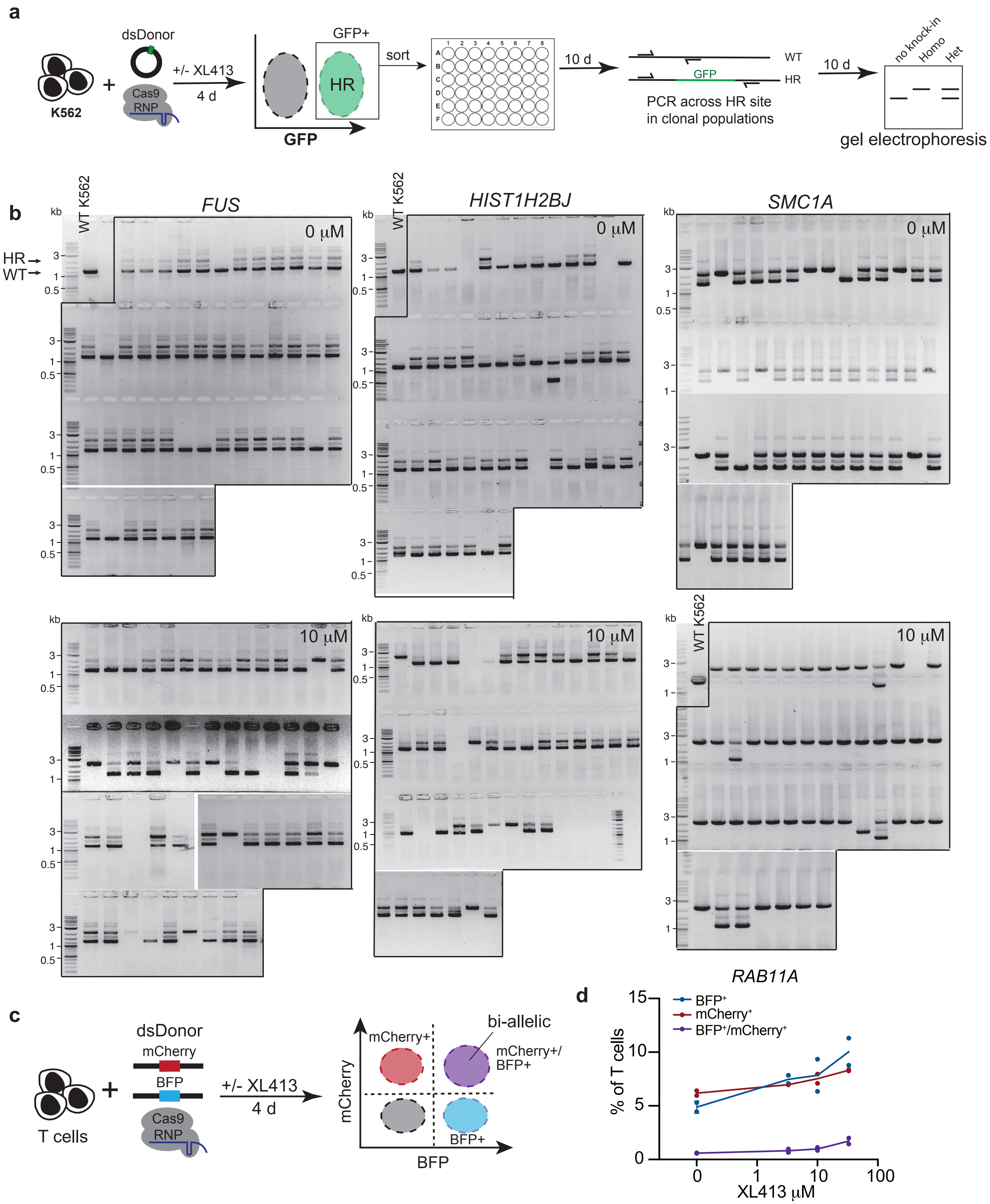

Supplementary Figure 5: Measurement of knock-in zygosity. **(a)** Workflow in K562 cells to test for homozygous knock-in. Cells were nucleofected with RNPs targeting the indicated loci with dsDonors containing a C-terminal *GFP* fusion to the respective protein and treated with XL413 as indicated. At day 4, GFP^{+} cells were individually sorted into single-cell clones in 96-well plates, grown for 10 days, and individual alleles at the edited locus were quantified by PCR across the HR site. Clones were assigned a genotype based on the presence of only short PCR bands (sorting error of wild-type cells or non-targeted GFP expression), short and long PCR bands (heterozygous GFP tagging), or only long PCR bands (homozygous GFP tagging). **(b)** Agarose DNA gels used to assess genotypes in **[Fig. 5a]**. **(c)** Schematic showing biallelic editing in T cells. T cells were edited with RNPs targeting the *RAB11A* locus and two dsDonor templates encoding either mCherry or BFP and post-treated with the indicated concentrations of XL413. Single- or double-fluorescent reporter cell populations are quantified by flow cytometry. **(d)** Reproduction of data from **[Fig. 5b]** in the context of monoallelic editing rates. Values are shown as mean±SD (n=2 biological replicates).

Supplementary Figure 6

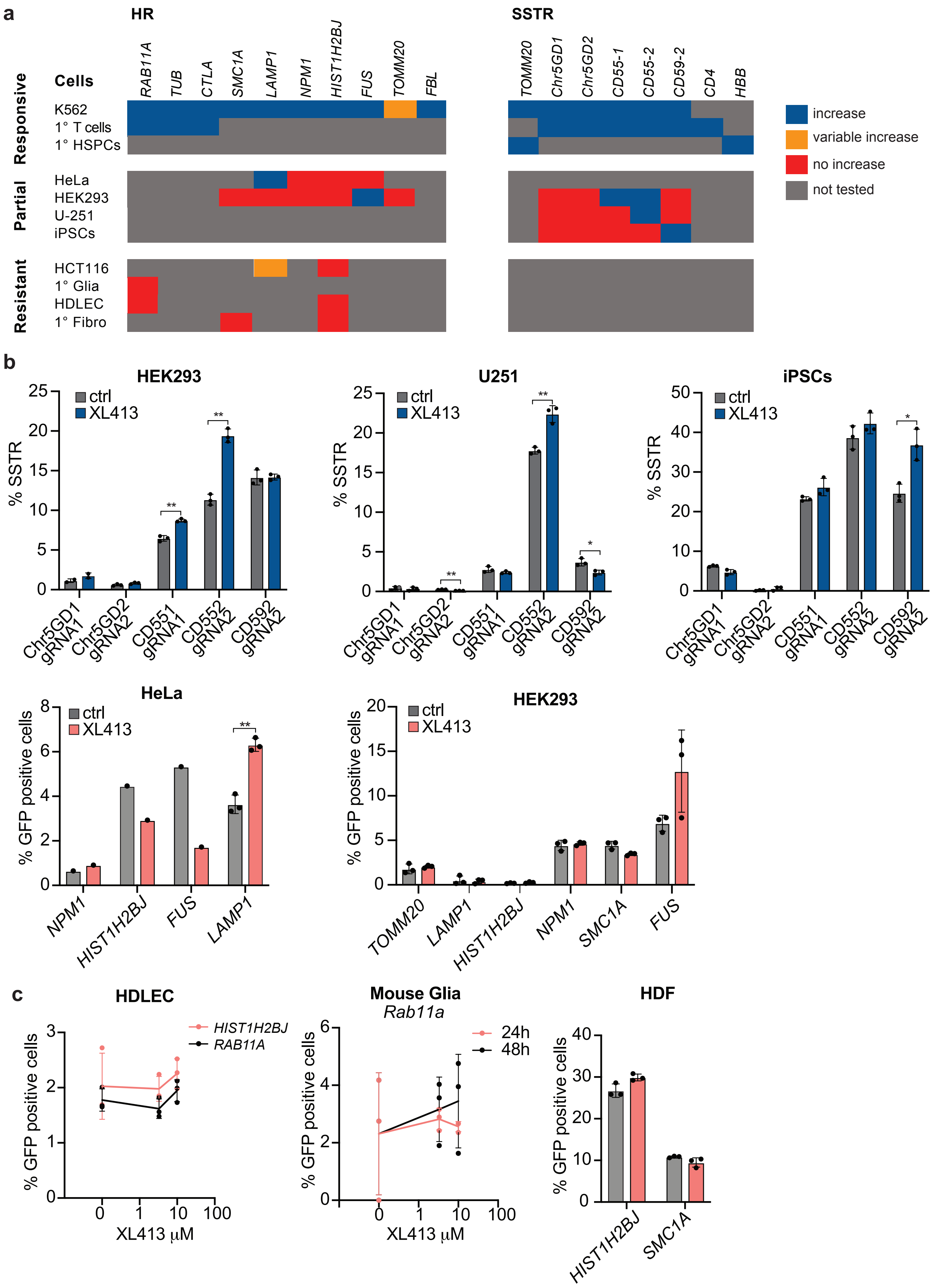

Supplementary Figure 6: XL413 stimulates HDR in a cell-type dependent manner. **(a)** Summary of XL413 experiments. XL413 was tested for its ability to stimulate HR, SSTR, or both in K562s, primary human T cells, primary human HSPCs, HeLa, HEK293T, U-251, iPSCs, HCT116, mouse primary glia, human primary HDLEC cells, and human primary dermal fibroblasts (HDFs). Green – increase in HDR; Yellow –variable increases; Red – no increase; Grey – not tested. Cells can be separated into three categories: Responsive to XL413, Partially responsive to XL413, and Resistant to XL413. **(b)** Data for partially responsive cell types showing rates of SSTR as measured by amplicon sequencing (HEK293, U251, iPSC) or rates of HR as determined by GFP fusion protein expression measured by flow cytometry (HeLa, HEK293). (**c**) Data for cell types that do not exhibit improved HR with XL413. All values are shown as mean±SD (biological replicates as indicated). Statistical significances were calculated by unpaired two-tailed t-test using the Holm-Sidak method (p-values are reported as *$p<0.05$, **$p<0.01$, ***$p<0.001$, ****$p<0.0001$).

Supplementary Figure 7

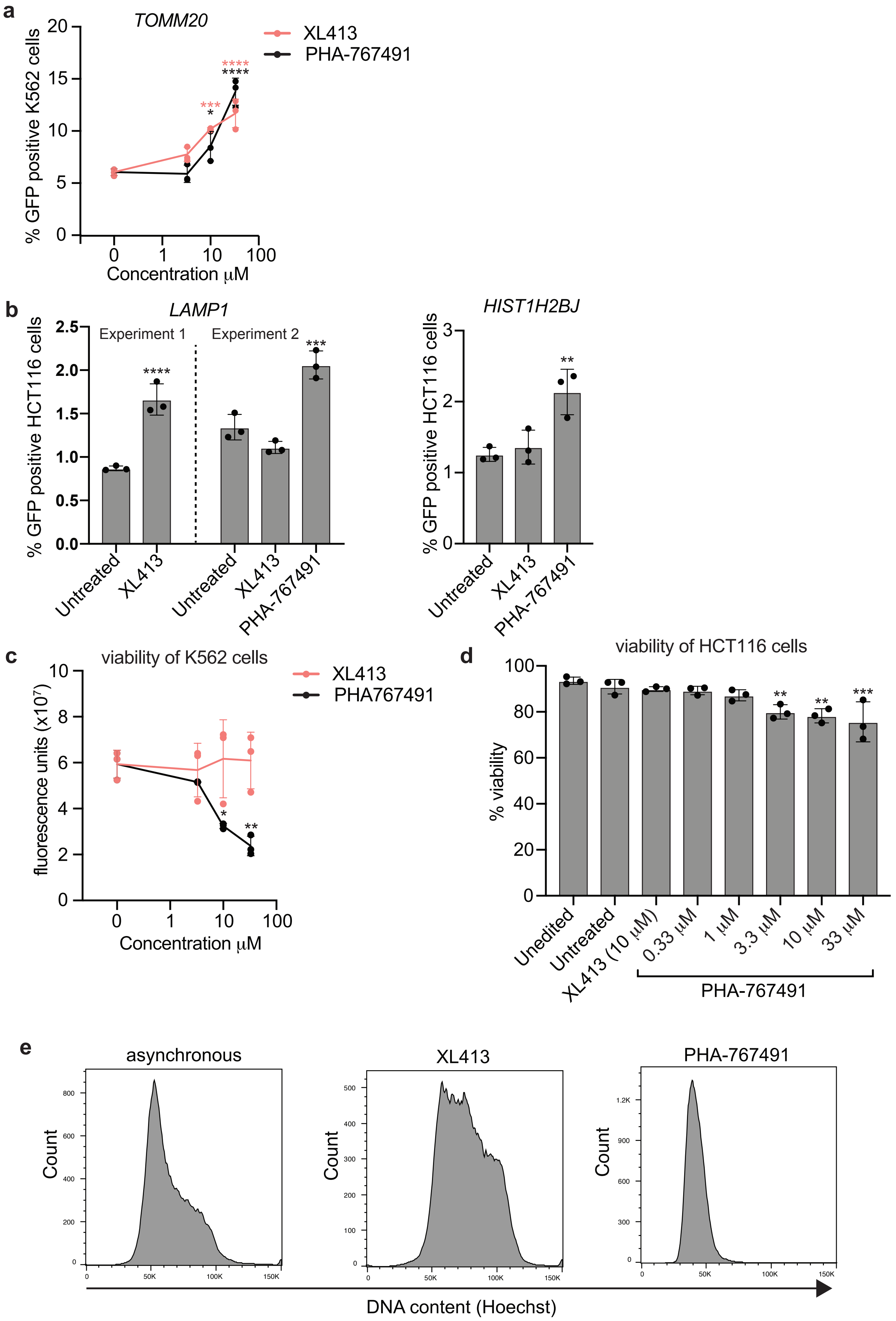

Supplementary Figure 7: Alternate forms of CDC7 inhibition can be effective HR enhancers in XL413-resistant cell lines. **(a)** XL413 and PHA-767491 both boost HR. K562 cells were nucleofected with RNPs targeting the *TOMM20* locus with a dsDonor encoding a C-terminal GFP fusion. Nucleofected cells were cultured in the indicated concentrations of XL413 or PHA-767491, and HR was quantified by flow cytometry 4 days after nucleofection. **(b)** PHA-767491 boosts HR in cells not responsive to XL413. HCT116 cells were nucleofected with RNP targeting the *HIST1H2BJ* or *LAMP1* genes with a dsDonor encoding a C-terminal fusion to GFP. Nucleofected cells were cultured in XL413 (10 μM) or PHA-767491 (1 μM) for 24 h, and HR indicated by GFP expression was quantified by flow cytometry 4 days after nucleofection. **(c)** PHA-767491 is more toxic than XL413 in K562 cells. Viability was determined 4 days after nucleofection using a Presto Blue viability assay, in which the total fluorescence produced by viable cells serves as a direct measure of cell viability. Cells treated with PHA-767491 showed significantly reduced viability. **(d)** Viability of HCT116 cells 4 days after nucleofection with RNPs and dsDonor then treated for 24 h with either XL413 or PHA-767491. Viability was determined and quantified by flow cytometry using characteristic FSC and SSC criteria. (**e**) PHA-767491 treatment results in a cell cycle arrest in G1 while treatment with XL413 results in a S-phase arrest. K562 cells were treated with each drug (10 μM) for 24 h and then stained with Hoechst33342 and analyzed by flow cytometry. All values are shown as mean±SD (n=3 biological replicates). Statistical significances were calculated by one-way ANOVA and Dunnet's multiple comparison test (adjusted p-values are reported as *$p<0.05$, **$p<0.01$, ***$p<0.001$, ****$p<0.0001$).

Supplementary Figure 8

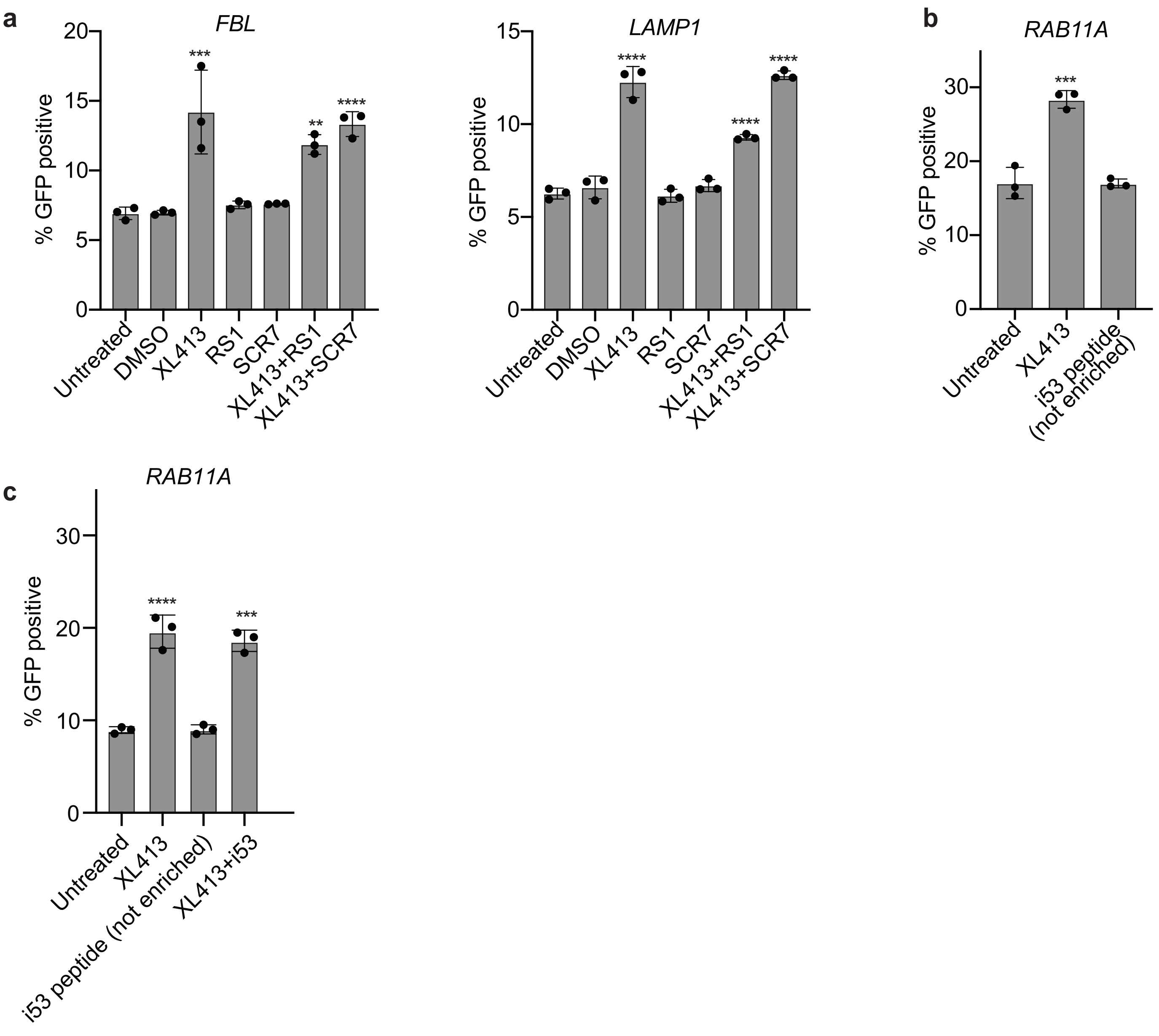

Supplementary Figure 8: CDC7 inhibition does not synergize with other HDR boosting treatments. **(a)** Effect of XL413 (33 μM), SCR7 (1 μM) and RS-1 (10 μM) treatment on editing outcomes in K562 cells. Cells were nucleofected with RNPs and plasmid dsDonor targeting GFP fusion to *FBL* or *LAMP1* loci, and small molecules were added to the media of nucleofected cells for 24h. Cells were analyzed by flow cytometry 4 days post nucleofection. **(b)** HR stimulation by i53 is dependent on transfection efficiency. Data presented as described in **[Fig. 6c]**, except i53-treated samples shown as the bulk population and not gating for i53-expressing cells. **(c)** i53 does not synergize with XL413 treatment. Cells were nucleofected with RNPs and a GFP plasmid dsDonor targeting the *RAB11A* locus in the setting of pre-treatment with i53, post-treatment with XL413 for 24 hours, or both pre-treated with i53 and post-treated with XL413. Cells were analyzed by flow cytometry 4 days post nucleofection. Values in panels a-c are shown as mean±SD (n=3 biological replicates). All values are shown as mean±SD (n=3 biological replicates). Statistical significances were calculated by one-way ANOVA and Dunnet's multiple comparison test (adjusted p-values are reported as *p<0.05, **p<0.01, ***p<0.001, ****p<0.0001).

Supplementary Figure 9

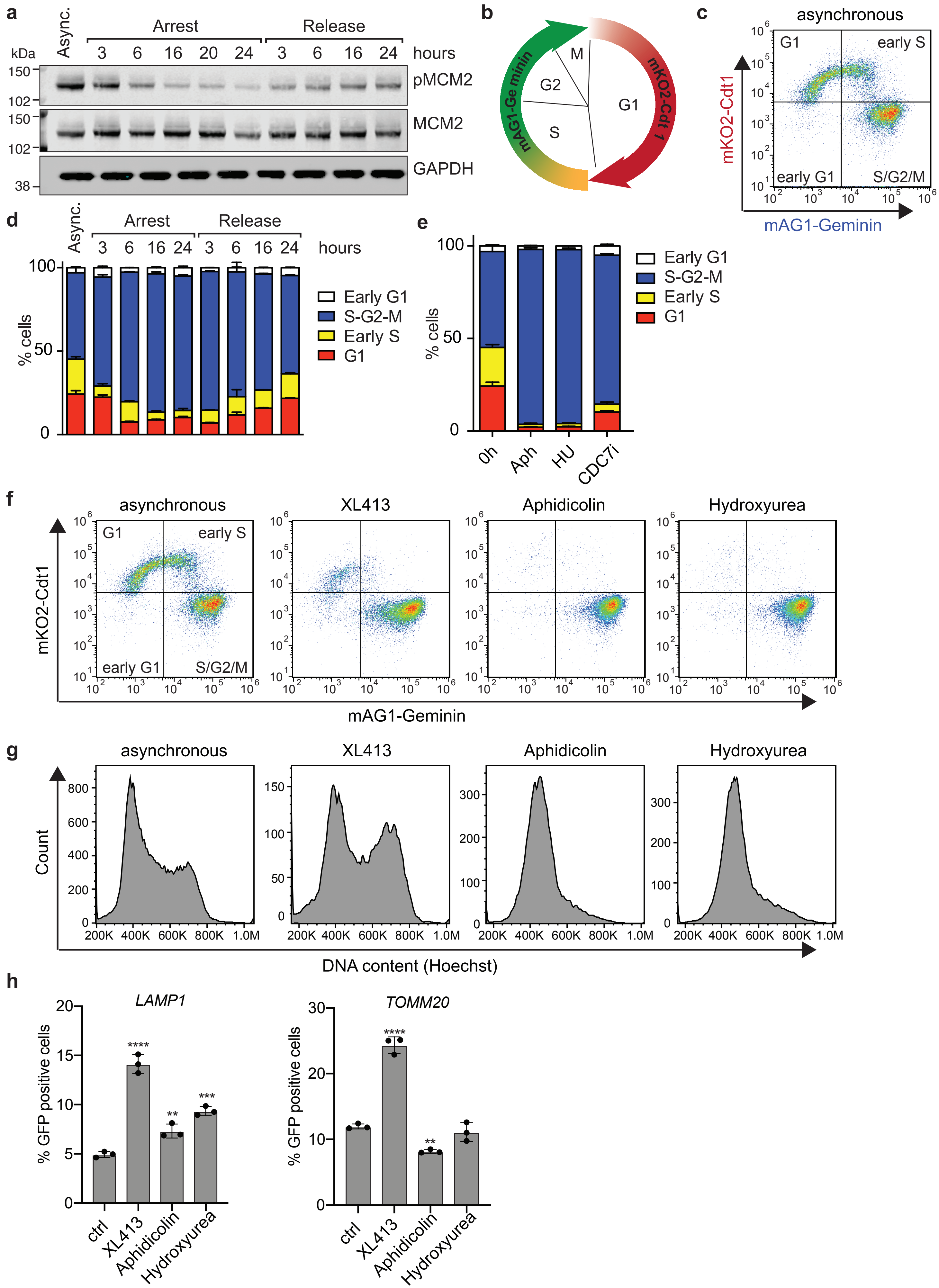

Supplementary Figure 9: CDC7 inhibition promotes reversible cell cycle arrest. (**a**) CDC7 inhibition reduces phospho-MCM2 by western blot. K562 cells were either left asynchronous or treated with XL413. Cells were harvested for protein extraction at indicated time points during arrest-release experiments. Shown is a representative experiment (repeated once with similar results). **(b)** Schematic showing the cell cycle dynamics of the FUCCI system[39]. (**C**) Flow cytometry of asynchronous K562-FUCCI cells with indicated cell cycle phases. (**D**) CDC7 arrest alters cell cycle phase dynamics. Flow cytometry profile of K562-FUCCI cells that were treated with XL413 (33 μM) for 24 h and then released into media without XL413. **(E-F)** Cell cycle phase profiles measured by flow cytometry of arrested K562-FUCCI cells. CDC7 arrest (XL413 33 μM) is distinct from arrests produced by Aphidicolin (2 μg mL^$^{-1}$) or Hydroxyurea (2 mM), as measured by the FUCCI reporter system or **(G)** Hoechst33342 staining for DNA content. **(H)** XL413 treatment, but not Aphidicolin or Hydroxyurea treatment, consistently boosts HR rates in K562 cells. Cells were nucleofected with RNPs targeting the *LAMP1 or TOMM20* locus with a plasmid dsDonor encoding a *GFP* fusion at the C-terminus, and small molecules were added to the media of nucleofected cells for 24 h (XL413 (33 μM), Aphidicolin (2 μg mL^$^{-1}$) or Hydroxyurea (2 mM)). Cells were analyzed by flow cytometry 4 days post nucleofection. All values are shown as mean±SD (n=3 biological replicates). Statistical significances were calculated by one-way ANOVA and Dunnet's multiple comparison test (adjusted p-values are reported as *$p<0.05$, **$p<0.01$, ***$p<0.001$, ****$p<0.0001$).
