# Supplementary material for: Timed inhibition of CDC7 increases CRISPR-Cas9 mediated templated repair
Source: Nat Commun. 2020 Apr 30;11:2109. doi: 10.1038/s41467-020-15845-1 (PMC7193628; doi:10.1038/s41467-020-15845-1)
Supplement: Supplementary file 5 — Reporting Summary [file 41467_2020_15845_MOESM5_ESM.pdf]

nature research

Corresponding author(s): Corn and Richardson

Last updated by author(s): Mar 8, 2020

Reporting Summary

Nature Research wishes to improve the reproducibility of the work that we publish. This form provides structure for consistency and transparency in reporting. For further information on Nature Research policies, see Authors & Referees and the Editorial Policy Checklist .

Statistics

For all statistical analyses, confirm that the following items are present in the figure legend, table legend, main text, or Methods section.

n/a	Confirmed	
☐	☒	The exact sample size (*n*) for each experimental group/condition, given as a discrete number and unit of measurement
☐	☒	A statement on whether measurements were taken from distinct samples or whether the same sample was measured repeatedly
☐	☒	The statistical test(s) used AND whether they are one- or two-sided<br>*Only common tests should be described solely by name; describe more complex techniques in the Methods section.*
☐	☒	A description of all covariates tested
☐	☒	A description of any assumptions or corrections, such as tests of normality and adjustment for multiple comparisons
☐	☒	A full description of the statistical parameters including central tendency (e.g. means) or other basic estimates (e.g. regression coefficient) AND variation (e.g. standard deviation) or associated estimates of uncertainty (e.g. confidence intervals)
☐	☒	For null hypothesis testing, the test statistic (e.g. *F*, *t*, *r*) with confidence intervals, effect sizes, degrees of freedom and *P* value noted<br>*Give P values as exact values whenever suitable.*
☒	☐	For Bayesian analysis, information on the choice of priors and Markov chain Monte Carlo settings
☒	☐	For hierarchical and complex designs, identification of the appropriate level for tests and full reporting of outcomes
☒	☐	Estimates of effect sizes (e.g. Cohen's *d*, Pearson's *r*), indicating how they were calculated

Our web collection on statistics for biologists contains articles on many of the points above.

Software and code

Policy information about availability of computer code

Data collection	Cytometry - Attune cytometer (v3.1); Blots - LiCor Image Studio (v5); qPCR - BioRad CFX96, Excel 16.33; Amplicon sequencing - Illumina Miseq; Qubit 3.0
Data analysis	FACS - flowjo (v10); Pooled screen - https://doi.org/10.7554/eLife.19760.001; Amplicon sequencing - cortado (v1.0); Pathway analysis - DAVID (v6.8); Graphing - Prism (v8.4);

For manuscripts utilizing custom algorithms or software that are central to the research but not yet described in published literature, software must be made available to editors/reviewers. We strongly encourage code deposition in a community repository (e.g. GitHub). See the Nature Research guidelines for submitting code & software for further information.

Data

Policy information about availability of data

All manuscripts must include a data availability statement. This statement should provide the following information, where applicable:

- Accession codes, unique identifiers, or web links for publicly available datasets
- A list of figures that have associated raw data
- A description of any restrictions on data availability

Sequencing reads used in the pooled screen and amplicon sequencing data were deposited in SRA (PRJNA610420).

Field-specific reporting

Please select the one below that is the best fit for your research. If you are not sure, read the appropriate sections before making your selection.

☒ Life sciences ☐ Behavioural & social sciences ☐ Ecological, evolutionary & environmental sciences

For a reference copy of the document with all sections, see nature.com/documents/nr-reporting-summary-flat.pdf

Life sciences study design

All studies must disclose on these points even when the disclosure is negative.

Sample size	No calculations were performed to determine sample size.
Data exclusions	No data was excluded. Technical failure (microbial contamination, failed siRNA knockdown, etc) were predefined criteria for rejection.
Replication	Cells were separated into three stocks, then nucleofected, recovered, and analyzed separately (biological triplicate). All data replicated and are shown in manuscript.
Randomization	No randomization was performed.
Blinding	Investigators were not blinded to experimental outcomes.

Reporting for specific materials, systems and methods

We require information from authors about some types of materials, experimental systems and methods used in many studies. Here, indicate whether each material, system or method listed is relevant to your study. If you are not sure if a list item applies to your research, read the appropriate section before selecting a response.

Materials & experimental systems

n/a	Involved in the study	
☐	☒	Antibodies
☐	☒	Eukaryotic cell lines
☒	☐	Palaeontology
☒	☐	Animals and other organisms
☐	☒	Human research participants
☒	☐	Clinical data

Methods

n/a	Involved in the study	
☒	☐	ChIP-seq
☐	☒	Flow cytometry
☒	☐	MRI-based neuroimaging

Antibodies

Antibodies used	FLAG (Sigma F1804, 1:1000), TOMM20 (Cell Signaling 42406, 1:1000), GAPDH (cell Signaling 97166, 1:5000), phospho-S53 MCM2 (Abcam ab109133, 1:1000), MCM2 (Abcam ab6153, 1:1000), 1:10,000 donkey anti-mouse IgG-IR800 (Li-Cor 925-32212), 1:10,000 donkey anti-mouse IgG-IR680 (Li-Cor 925-68022), 1:10,000 donkey anti-rabbit IgG-IR800 (Li-Cor 925-32213), 1:10,000 donkey anti-rabbit IgG-IR680 (Li-Cor 925-68023)
Validation	Antibodies were purchased new and used per manufacturer's advice. No further validation was performed.

Eukaryotic cell lines

Policy information about cell lines

Cell line source(s)	K562, HEK293, HDLECs, HCT116s, HeLa, and U251 cells were acquired from ATCC or the Berkeley Cell Culture Facility.
Authentication	STR Profiling
Mycoplasma contamination	All lines tested negative and are routinely retested.
Commonly misidentified lines (See ICLAC register)	None of the cell lines in this study are listed in the ICLAC database.

Human research participants

Policy information about studies involving human research participants

Population characteristics	Primary human T cells were isolated from two de-identified healthy human donors
Recruitment	T-cells were residuals from leukoreduction chambers after Trima Apheresis (Vitalant)
Ethics oversight	UCSF IRB (BU101283)

Note that full information on the approval of the study protocol must also be provided in the manuscript.

Flow Cytometry

Plots

Confirm that:

- [x] The axis labels state the marker and fluorochrome used (e.g. CD4-FITC).
- [x] The axis scales are clearly visible. Include numbers along axes only for bottom left plot of group (a 'group' is an analysis of identical markers).
- [x] All plots are contour plots with outliers or pseudocolor plots.
- [x] A numerical value for number of cells or percentage (with statistics) is provided.

Methodology

Sample preparation	Cells were minimally processed (resuspended in standard media)
Instrument	Attune NxT, Sony SH800.
Software	FlowJo v10
Cell population abundance	Analytical flow had >5000 cells. Pooled screen had >7,000,000 total cells.
Gating strategy	Cells populations were identified with a FSC-A/SSC-A gate. Singlets were identified using a SSC-A/SSC-H gate. Viability was confirmed by Trypan blue staining (cell lines) or GhostDye780 (T-cells). Other analyses were performed on the single cell population (cell lines) or the viable single cell population (T-cells).

- [x] Tick this box to confirm that a figure exemplifying the gating strategy is provided in the Supplementary Information.
