# Supplementary material for: Timed inhibition of CDC7 increases CRISPR-Cas9 mediated templated repair
Source: Nat Commun. 2020 Apr 30;11:2109. doi: 10.1038/s41467-020-15845-1 (PMC7193628; doi:10.1038/s41467-020-15845-1)
Supplement: Supplementary file 6 — Description of Additional Supplementary Files [file 41467_2020_15845_MOESM6_ESM.pdf]

Title: Supplementary Data 1
Pooled screen results

Title: Supplementary Data 2
NGS amplicon sequencing results

Title: Supplementary Data 3
All molecular biology reagents and reagent/gene lists
